# A Systematic Review of Population-Based Studies of Chronic Bowel Symptoms in Cancer Survivors following Pelvic Radiotherapy

**DOI:** 10.3390/cancers15164037

**Published:** 2023-08-09

**Authors:** Adam Biran, Iakov Bolnykh, Ben Rimmer, Anthony Cunliffe, Lisa Durrant, John Hancock, Helen Ludlow, Ian Pedley, Colin Rees, Linda Sharp

**Affiliations:** 1 Centre for Cancer, Population Health Sciences Institute, Newcastle University, Newcastle upon Tyne NE1 7RU, UK; iakov.bolnykh@newcastle.ac.uk (I.B.); ben.rimmer@newcastle.ac.uk (B.R.); colin.rees@newcastle.ac.uk (C.R.); linda.sharp@newcastle.ac.uk (L.S.); 2 NHS Southwest London Clinical Commissioning Group, London SW19 1RH, UK; anthony.cunliffe@nhs.net; 3 Somerset NHS Foundation Trust, Taunton TA1 5DA, UK; lisa.durrant@somersetft.nhs.uk; 4 North Tees and Hartlepool NHS Foundation Trust, Hartlepool TS24 9AH, UK; j.hancock3@nhs.net; 5 Llandough, Cardiff and Vale University Health Board, Cardiff CF64 2XX, UK; helen.ludlow@wales.nhs.uk; 6 Newcastle upon Tyne Hospitals NHS Trust, Newcastle upon Tyne NE3 3HD, UK; ian.pedley@nhs.net

**Keywords:** radiotherapy, pelvic radiotherapy, pelvic radiation disease, chronic bowel symptoms, systematic review, radiotherapy late-effects, cancer survivorship, prostate cancer, gynecological cancer

## Abstract

**Simple Summary:**

Pelvic radiotherapy is used to treat a range of cancers. Radiotherapy can damage surrounding, non-cancerous tissue and organs, causing long-term problems, including bowel symptoms such as bleeding, pain, and incontinence. The provision of support and treatment for those affected as well as shared decision making regarding treatment should be informed by a solid understanding of the prevalence, nature, and severity of symptoms. We conducted a systematic review of population-based studies presenting patient-reported bowel symptoms to synthesize evidence on symptom prevalence and severity following pelvic radiotherapy. Multiple different bowel symptoms have been reported, and prevalence varies from 1% (bleeding) to 59% (anal bleeding for >12 months). We found substantial variation in the reported methods and few data pertaining to cancers other than prostate. Our review supports the view that bowel symptoms are a significant problem following pelvic radiotherapy and highlights limitations of the evidence base that should be addressed in future research.

**Abstract:**

Pelvic radiotherapy can damage surrounding tissue and organs, causing chronic conditions including bowel symptoms. We systematically identified quantitative, population-based studies of patient-reported bowel symptoms following pelvic radiotherapy to synthesize evidence of symptom type, prevalence, and severity. Medline, CINAHL, EMBASE, and PsychINFO were searched from inception to September 2022. Following independent screening of titles, abstracts, and full-texts, population and study characteristics and symptom findings were extracted, and narrative synthesis was conducted. In total, 45 papers (prostate, *n* = 39; gynecological, *n* = 6) reporting 19 datasets were included. Studies were methodologically heterogeneous. Most frequently assessed was bowel function (‘score’, 26 papers, ‘bother’, 19 papers). Also assessed was urgency, diarrhea, bleeding, incontinence, abdominal pain, painful hemorrhoids, rectal wetness, constipation, mucous discharge, frequency, and gas. Prevalence ranged from 1% (bleeding) to 59% (anal bleeding for >12 months at any time since start of treatment). In total, 10 papers compared radiotherapy with non-cancer comparators and 24 with non-radiotherapy cancer patient groups. Symptom prevalence/severity was greater/worse in radiotherapy groups and symptoms more common/worse post-radiotherapy than pre-diagnosis/treatment. Symptom prevalence varied between studies and symptoms. This review confirms that many people experience chronic bowel symptoms following pelvic radiotherapy. Greater methodological consistency, and investigation of less-well-studied survivor populations, could better inform the provision of services and support.

## 1. Introduction

The term pelvic radiation disease has been applied to a group of chronic symptoms that may arise following pelvic radiotherapy [1,2]. These symptoms arise through a variety of physiological routes, sharing the common trigger of exposure to pelvic radiation in adulthood, perhaps as treatment for cancer in the pelvic region, such as cervical or prostate cancer. Symptoms may include pain, fatigue, and skin changes, as well as sexual, urinary, and bowel problems. Bowel problems are often predominant [1] and may have a substantial negative impact on individuals’ (henceforth survivors’) overall quality of life (QoL) [3] and on their ability to perform occupational roles and participate in social activities [4]. These experiences also impact negatively on survivors’ families and friends [4].

Acute bowel symptoms often arise during treatment and, for many, resolve within three months of the cessation of radiotherapy [5]. Chronic symptoms may persist or arise for the first time beyond this period, sometimes years or decades after exposure [6]. However, temporal trends in prevalence or severity do not appear to have been systematically documented, despite this being key information to inform shared treatment decision making. Moreover, risk factors for experiencing chronic symptoms are poorly understood. The dose and site of radiation are likely to play a significant role; however, clinical (e.g., co-morbidities) and patient-related (e.g., age, lifestyle, and genetics) factors may also be involved [6].

Treatment options for post-radiotherapy bowel symptoms remain limited and are not without risk [7,8]. Novel treatments may be required, especially for survivors experiencing the most significant symptoms or those with the greatest impact. However, it is also likely that for some survivors, suffering could be alleviated through the holistic application of existing support and interventions if the cause of the symptoms was recognized, appropriate investigations and referrals were performed, and necessary support and treatment were provided [9,10]. A comprehensive understanding of the burden of chronic bowel problems after pelvic radiotherapy, and the survivor groups most likely to be affected, would be valuable to inform the planning and provision of supportive care services.

This systematic review aimed to identify data on chronic bowel symptoms reported by survivors following pelvic radiotherapy. Specific objectives were to synthesize data on symptom type, prevalence, and severity; temporal patterns in symptoms post-treatment; and survivor factors associated with increased prevalence and/or severity of symptoms. We focused on data from survivors recruited from population-based sampling frames to reduce the influence of selection biases on eligible studies.

## 2. Methods

This review was registered (CRD42021274083) with the Prospective Register for Systematic Reviews (PROSPERO) and conducted and reported in accordance with the Preferred Reporting Items for Systematic Review and Meta-Analysis (PRISMA) guidelines [11].

### 2.1. Information Sources and Search Strategy

Systematic searches were carried out in Medline, the Excerpta Medica Database (EMBASE), American Psychological Association (APA) PsychINFO, and the Cumulative Index of Nursing and Allied Health Literature (CINAHL). The search, developed with input from a medical librarian, covered three concepts: radiotherapy, pelvic cancer, and bowel symptoms (Supplementary Table S1). No date limits were applied. Final database searches were performed on 13 September 2022. Reference lists and forward citations of eligible papers and relevant reviews were hand-searched to identify any additional papers not previously retrieved.

### 2.2. Screening

After de-duplication, titles and abstracts were screened independently by two reviewers (reviewer one, AB, and reviewer two, IB or LS). Full texts of papers considered potentially eligible by reviewers one or two were obtained and assessed, again by two reviewers, against the inclusion criteria. Discrepancies were resolved by discussion, with the third reviewer (LS) consulted in the event of disagreement.

### 2.3. Eligibility Criteria

A paper was eligible if it was a primary, peer-reviewed research article, available in English which reported the results of a population-based quantitative study that included at least 100 survivors exposed to pelvic radiotherapy, this was to ensure focus on studies large enough to provide some confidence in precision of findings. Papers had to report the prevalence and/or severity of chronic symptoms (defined following Grodsky et al. as arising or lasting 3 months or more post-radiotherapy [5]) and the bowel symptoms had to be reported by survivors themselves.

### 2.4. Exclusion Criteria

A paper was excluded if (1) the study population was participating in a clinical trial, as survivors may have experienced more intensive follow-up or intervention than constitutes routine care; (2) bowel symptoms were based on data abstracted from clinical records or clinician’s assessment; (3) outcomes were not collected in a standardized way across all participants; (4) it reported qualitative results only or a case study; (5) the study included survivors treated with different modalities, and it was not possible to disaggregate the results for irradiated survivors; (6) it was not possible to disaggregate chronic from acute symptoms; (7) the study was of colorectal cancer survivors due to the problem of distinguishing effects of radiotherapy from the effects of the cancer and/or associated surgery on the bowel; or (8) data for small sub-groups of irradiated survivors could not be readily combined to achieve a sample size ≥ 100. Criteria 1–6 were specified a priori, and criteria 7 and 8 were added following initial scoping searches. 

### 2.5. Data Extraction and Quality Appraisal 

Data extraction from eligible papers was conducted by one reviewer onto a structured form and checked by another (AB and IB) with discrepancies resolved by discussion. We extracted data describing study characteristics (e.g., design, sample size), population characteristics (e.g., cancer and treatment details), and findings (i.e., data describing bowel symptom prevalence and/or severity in survivors exposed to pelvic radiotherapy for all time-points reported; results of multivariate (if available) or univariate comparisons with groups not exposed to pelvic radiotherapy (i.e., non-cancer or other treatment groups); and any comparisons of prevalence/severity between subgroups defined by survivor characteristics).

Quality appraisal used an adapted 10-item version of the Methodological Index for Non-Randomized Studies (MINORS) tool [12]. Each item was scored zero (not reported), one (partially reported), or two (fully reported). Maximum possible scores were 14 for non-comparative studies and 20 for studies with a non-radiotherapy or non-cancer comparator group. Following previous authors [13], studies scoring ≥ 14 were considered ‘high quality’. Non-comparative studies had to have the maximum possible score to be considered high quality. Each paper was appraised by one reviewer (AB or IB) and cross-checked by the other, with discrepancies resolved through discussion with a third reviewer (LS) if necessary. If a paper referred to earlier methods papers, those were also consulted, and relevant information was extracted.

### 2.6. Data Synthesis

Papers reporting results from survivors drawn from the same data source(s) and treated/diagnosed during the same period were regarded as a single study reporting on the same dataset. Papers using the same data source but covering different periods of diagnosis/treatment were considered separate studies.

Heterogeneity with respect to cancer sites, symptoms assessed, data collection tools, and assessment time points precluded meta-analysis. Instead, narrative synthesis was undertaken [14]. We present an overview of the synthesized findings followed, to be comprehensive, by a synthesis of the findings by symptom.

## 3. Results

### 3.1. Search Results and Characteristics of Eligible Papers

**Figure 1:**
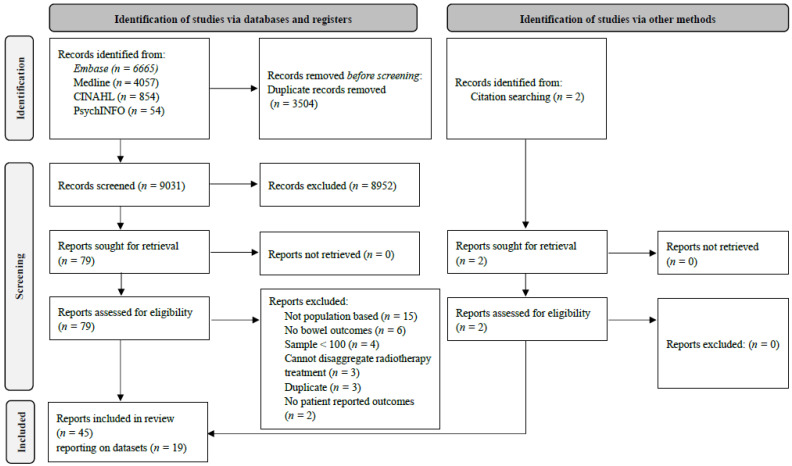
PRISMA flow diagram.

Following de-duplication, 9031 citations underwent title and abstract screening (Figure 1); 79 were selected for full-text review, of which 43 were eligible. Hand searches identified two additional eligible citations. Therefore, 45 papers from 19 datasets were included [15,16,17,18,19,20,21,22,23,24,25,26,27,28,29,30,31,32,33,34,35,36,37,38,39,40,41,42,43,44,45,46,47,48,49,50,51,52,53,54,55,56,57,58,59]. Characteristics of individual papers, and associated populations, are presented in Supplementary Tables S2 and S3, respectively.

Papers reported on datasets from nine countries: USA (*n* = 5 datasets; *n* = 20 papers) [16,20,21,28,29,30,31,32,33,35,40,43,48,49,50,52,53,56,57,59], Australia (*n* = 4 datasets of which *n* = 1 reported with New Zealand; *n* = 5 papers) [17,38,44,51,54]; the Netherlands (*n* = 2 datasets; *n* = 2 papers) [39,58], Sweden (*n* = 2 datasets; *n* = 6 papers) [15,18,19,24,25,55], the United Kingdom (*n* = 2 datasets; *n* = 6 papers) [22,37,42,45,46,47], Denmark (*n* = 1 dataset; *n* = 1 paper) [41], Ireland and Northern Ireland (*n* = 1 dataset reported together; *n* = 2 papers) [23,27], Finland (*n* = 1 dataset; *n* = 1 paper) [36], and Norway (*n* = 1 dataset; *n* = 2 papers) [26,34]. A total of 39 papers reported findings from 16 datasets of prostate cancer survivors [16,17,19,20,21,22,23,26,27,28,29,30,31,32,33,34,35,36,37,38,39,40,41,42,43,44,45,46,47,48,49,50,51,52,53,54,56,57,59] and 6 papers from 3 datasets of gynecological cancer survivors (endometrial *n* = 1 [58], cervical *n* = 1 [18], mixed gynecological cancers *n* = 4 [15,24,25,55]).

In total, 22 papers (*n* = 12 datasets) reported cross-sectional analyses [15,18,19,22,23,24,25,26,27,29,30,34,36,39,42,44,45,46,47,51,55,58] and 23 papers (*n* = 9 datasets; all prostate) reported longitudinal analyses [16,17,20,21,28,31,32,33,35,37,38,40,41,43,48,49,50,52,53,54,56,57,59]. Seven different previously validated outcome measurement tools were used (Supplementary Table S4): Expanded Prostate Cancer Index Composite (EPIC)-26 (*n* = 23 papers) [16,17,21,22,26,30,31,32,35,37,38,39,41,42,43,44,45,46,47,51,56,57,59], University of California Los Angeles Prostate Cancer Index (UCLA PCI) (*n* = 4) [30,38,43,54], Prostate Cancer Symptoms Indices (PCSI) (*n* = 2) [20,40], European Organization for Research and Treatment of Cancer (EORTC), Quality of Life Questionnaire (QLQ)-30 (*n* = 2) [23,27], EORTC QLQ-Prostate Cancer (PR)25 (*n* = 2) [23,27], EORTC QLQ-Endometrial Cancer (EN)24 (*n* = 1) [58], and EPIC-50 (*n* = 1) [34]. Thirteen papers used either a study-specific questionnaire or adapted an existing questionnaire [15,18,19,24,25,28,33,36,49,50,52,53,55].

The most frequently reported bowel outcome was a bowel function score (*n* = 26 papers) [16,20,21,22,23,26,28,31,32,37,38,39,40,41,42,43,44,45,46,47,51,54,56,57,58,59] followed by bowel function ‘bother’ (*n* = 19) [15,16,17,19,21,26,27,28,30,31,33,34,35,48,49,51,52,53,54], urgency (*n* = 16) [15,16,18,21,22,25,26,28,29,31,35,48,49,50,52,55], bleeding (*n* = 10) [15,16,21,25,26,31,36,48,50,55], fecal leakage/incontinence (*n* = 8) [15,18,24,25,26,34,53,55], diarrhea (*n* = 6) [23,27,34,49,50,58], abdominal pain (*n* = 5) [18,26,28,49,50], painful hemorrhoids (*n* = 3) [28,49,50], rectal wetness (*n* = 3) [28,49,50], constipation (*n* = 2) [18,23], mucous discharge (*n* = 2) [50,55], frequency (*n* = 1) [28], and gas (*n* = 1) [55].

### 3.2. Quality Appraisal

Nine papers (all prostate, *n* = 8 with a comparison group) were scored as high-quality papers [16,20,29,33,38,49,50,53,54] (Table S5). Papers scored most poorly on reporting a priori sample size calculations and loss to follow-up.

### 3.3. Symptom Prevalence Cross-Sectionally and over Time

The timing of post-radiotherapy data collection varied across papers and ranged from 6 months (the most reported post-treatment time point) to 15 years. The prevalence of symptoms six months post-treatment/diagnosis ranged from 1% for bleeding [31] to 46% for bowel movement frequency (>3 times daily) [28] (Table 1).

All ten papers [21,28,29,31,33,35,48,53,54,59] which compared bowel symptoms post-radiotherapy to pre-radiotherapy reported a higher prevalence or greater severity post-treatment than beforehand (Table 2).

Overall, papers suggested a peak in the prevalence of most symptoms at six months (compared with baseline) and some decrease thereafter (Table 1). Exceptions to this were one study reporting the bowel symptom ‘bother’ [31] which found an increase in prevalence from 6 months to 1 year followed by a decrease, and one study reported painful hemorrhoids [28] which found higher prevalence at two years compared with six months post-treatment. A decrease over time to below-baseline levels was reported for ‘moderate/big’ bowel problems in one study [33] and for bleeding in one study [31]. One study [53] found no change from the baseline in prevalence of incontinence (defined by the authors as fecal leakage more than a few drops). All other studies reporting relevant data found that prevalence remained higher than the baseline across the timepoints for which data were presented. Post-treatment, there were five longitudinal studies reporting prevalence of at least one symptom at two or more time points [17,28,31,33,52]. Across these studies there were seven reports of decreasing prevalence of a symptom, four reports of an increasing prevalence, and one report of no change. For the two symptoms reported at more than one post-treatment time point in more than one study (general bowel symptoms and bleeding), the results were inconsistent across studies. Additionally, three longitudinal studies reporting bowel symptom scores at more than one time point post-treatment showed a decrease in severity at 24 months [17,28,31].

### 3.4. Comparisons with Non-Irradiated Populations

Five papers (*n* = 2 prostate; *n* = 3 gynecological) reported comparisons of irradiated populations with non-cancer comparators [19,24,25,26,55] and 25 (*n* = 2 gynecological) with a non-radiotherapy group (surgery or no treatment) diagnosed with the same cancer as the irradiated group [16,18,20,21,22,23,27,29,30,31,32,34,37,38,39,40,41,49,50,52,53,54,56,57,58]. All studies, bar one, reported a higher prevalence (or greater severity) of symptoms in the radiotherapy group (Table 2). The only exception was a finding (for which no statistical test was reported) that constipation was more prevalent among gynecological cancer survivors treated with surgery than those treated with radiotherapy [18].

### 3.5. Survivor Characteristics

Three papers from three US prostate datasets [33,53,56] compared bowel symptoms across ethnic groups. Two compared radiotherapy against other treatments. Schwartz et al. [53] reported that the odds of bowel incontinence following radiotherapy versus radical prostatectomy were higher among white than African American survivors; the analysis included <100 African American survivors. Tyson et al. [56] reported a statistically significant decline in bowel function among non-Hispanic white survivors treated with radiotherapy compared with active surveillance but not for African American or Hispanic groups. When comparing radiotherapy with radical prostatectomy, the paper reported a statistically significant decline in bowel function for non-Hispanic white and African American radiotherapy groups but not for Hispanic survivors. None of these differences were clinically significant. Johnson et al. [33] compared urgency over time (6 to 60 months) by ethnic group, reporting higher prevalence among non-Hispanic white than African American or Hispanic survivors at all time points, but this was only statistically significant versus Hispanic survivors at 6 months and African American survivors at 60 months. No differences in the bowel function score were found. Analyses include <100 non-White survivors who underwent radiotherapy.

No other comparisons of prevalence/severity or time trends by survivor characteristics were reported.

### 3.6. Comparison of Radiotherapy Treatments

Eight papers (all prostate, seven of which reported bowel function score) [42,43,44,45,46,47,48,59] presented comparisons of symptoms following different radiotherapy treatments. O’Neil et al. [43] found better bowel function in those treated with contemporary, intensity modulated radiotherapy when compared to those treated during the 1990s with standard external beam radiotherapy (EBRT). The other six papers reporting bowel function score found no difference comparing conventionally fractionated with hypofractionated radiotherapy [42]; prostate-only radiotherapy with pelvic radiotherapy [59]; pelvic-only radiotherapy with pelvic and lymph node radiotherapy [45]; and external beam radiotherapy with or without brachytherapy [44,46,47]. Pasalic et al. [48] compared external radiotherapy groups with or without additional brachytherapy on three outcomes: bowel bother, urgency, and bloody stools. A lower prevalence of bloody stools was reported in the group receiving additional brachytherapy. There were no differences in other outcomes.

### 3.7. Findings by Symptom

A summary of findings by symptom is presented in Supplementary Table S6.

#### 3.7.1. Bowel Function

Bowel function was reported as a score or a prevalence (sometimes at a stated degree of bother or problem) of experiencing bowel dysfunction.

Bowel function score: Twenty-six papers (4 high-quality papers; 13 datasets: 12 prostate and 1 gynecological) reported on bowel function score [16,20,21,22,23,26,28,31,32,37,38,39,40,41,42,43,44,45,46,47,51,54,56,57,58,59]. It was most frequently measured using EPIC-26 (*n* = 20 papers) [16,21,22,26,30,31,32,37,38,39,41,42,43,44,45,46,47,56,57,59], where scores <100 indicate impaired function. Mean EPIC-26 scores at ≥6 months post-RT ranged from 83.8 (18–42 months post-diagnosis [22]) to 90.3 (3 years post-diagnosis [16]).

Two papers (both prostate; one of a high quality) [21,28] presented statistical comparisons of bowel function pre- and post-treatment. Both found statistically significant poorer bowel function post-treatment in survivors receiving radiotherapy. Smith et al. [54] found poorer bowel function post-treatment compared with recalled function pre-diagnosis; no statistical tests were presented.

Fossa et al. [26] compared irradiated survivors with non-cancer comparators, finding statistically higher scores (worse symptoms) in the irradiated group. Twelve papers (three high-quality ones) from seven prostate datasets compared irradiated survivors with those managed in other ways [16,20,21,22,23,31,37,38,39,40,41,56]. Of these, eight papers from five datasets [16,21,22,37,38,39,41,56] reported statistically significantly worse scores in irradiated groups; two papers from two datasets [23,32] reported no statistical difference; and two papers from one dataset [20,40] reported statistically worse scores compared with an Active Surveillance group, for an EBRT group but not for a stereotactic body radiotherapy group [40], or for a brachytherapy group [20].

Van de Poll-Franse et al. [58] (gynecological cancer) reported statistically worse bowel function scores in a radiotherapy group than a group receiving other treatment(s).

#### 3.7.2. Bowel Problems Prevalence

In total, 19 papers (five high-quality ones) [15,16,17,19,21,26,27,28,30,31,33,34,35,48,49,51,52,53,54] from 11 datasets (10 prostate, 1 cervical) reported the prevalence of bowel problems or bowel dysfunction.

Seven papers from three prostate datasets compared pre- and post-treatment [21,28,31,33,35,48,53]. Of these, six reported higher prevalence post-treatment [21,28,31,33,35,48]; only Hamilton et al. [28] reported a statistically significant difference.

Fossa et al. [26] reported higher problem prevalence in a radiotherapy group than non-cancer comparators; statistical tests were not presented. Ten prostate papers compared radiotherapy with other treatments. Five, from three datasets, reported no statistical difference in the prevalence of bowel symptoms [16,31,35,49,53]. Five reported higher prevalence in a radiotherapy group, of which four (three datasets) presented statistical support [27,34,52,54].

Bergmark [18] reported higher prevalence in a radiotherapy cervical cancer group than a non-cancer group; no statistical test was reported.

#### 3.7.3. Urgency

Sixteen papers (four of a high quality) from six datasets (five prostate, one gynecological) reported on urgency [15,16,18,21,22,25,26,28,29,31,35,48,49,50,52,55]. Prevalence ranged from 4% [35] to 36% [52] for prostate cancer and from 29% (at least once per week in the last six months) [25] to 44% (any urgency distress) [18] for gynecological.

Three papers (one prostate; two gynecological from one dataset) [25,26,55] compared radiotherapy with non-cancer comparators. All found higher urgency prevalence in the radiotherapy group, which was statistically significant in the two studies reporting tests [25,55]. Eight prostate papers from five datasets compared radiotherapy with other treatments [16,21,22,29,31,49,50,52]. Two papers (one dataset) [16,31] reported no difference. The remaining six (four datasets) [21,22,29,49,50,52] reported higher prevalence in the radiotherapy group, which was significant in the five papers which reported statistical tests [21,22,49,50,52]. Similarly, the single gynecological cancer paper reporting a comparison [18] found a statistically higher prevalence of urgency in a radiotherapy group versus other treatments.

#### 3.7.4. Bleeding (Anal or Rectal Bleeding or Blood in Stools)

Ten papers (eight prostate, four datasets [15,16,21,26,31,36,48,50]; two gynecological, one dataset [25,55]) reported on bleeding. Prevalence (prostate) ranged from 1% [31,48] (moderate or big problem with bloody stools) to 59% (anal bleeding lasting more than 12 months at any time since start of treatment) [36] and (gynecological) from 7% (anal leakage of blood while awake at least occasionally) to 17% (rectal bleeding at least occasionally) [25].

Two gynecological papers from the same dataset [25,55] compared irradiated survivors with non-cancer comparators, finding bleeding (anal leakage of blood or bleeding syndrome) statistically more common in the irradiated group. Four prostate papers (two of a high quality) from two datasets [16,21,31,50] compared radiotherapy with no radiotherapy. Only one found evidence of higher bleeding prevalence at one year in irradiated as compared to surgically treated patients.

#### 3.7.5. Incontinence (Fecal Leakage or Unintended Emptying of Bowels)

Eight papers (one high-quality one) [15,18,24,25,26,34,53,55] from four datasets (three prostate, one gynecological) reported symptoms of incontinence (fecal leakage or unintended emptying of bowels). Prevalence of leakage ranged from 2% [53] to 14% [34] for prostate cancer survivors and 12% (‘empty bowels into clothing without warning’) to 33% (some leakage at least occasionally while awake) for gynecological cancer survivors [25]. The single paper which compared prevalence pre- and post-treatment (prostate, of a high quality) found statistically higher prevalence of incontinence outcomes (‘stool leakage’, ‘stool leakage more than a few drops and use of pads’) post-treatment [53].

All four papers (one prostate, three gynecological from the same dataset) which compared incontinence in irradiated survivors with non-cancer comparators reported higher prevalence in the cancer group. This was statistically significant in the three gynecological papers. Two prostate papers from different datasets, and one gynecological paper, reported higher prevalence of fecal incontinence in irradiated compared to non-irradiated patients. This was statistically significant in the prostate papers.

Two papers (one of a high quality; two datasets; prostate) [34,53] reported statistically higher prevalence of symptoms in a radiotherapy group compared with other treatments. One gynecological paper [18] reported higher prevalence of symptoms in a group treated with radiotherapy compared to a non-radiotherapy group (statistical tests not presented).

#### 3.7.6. Diarrhea

Seven papers (two of a high quality; six datasets; five prostate, one cervical, and one endometrial) [18,23,36,49,50,53,58] reported chronic diarrhea or loose stools post-radiotherapy. The prevalence was 14% [53] to 37% [49] among prostate survivors and 16% [58] to 42% [18] among gynecological survivors.

One prostate paper compared diarrhea prevalence pre- and post-radiotherapy and reported a statistically significant increase of 7.6 percentage points [53].

Three papers (two of a high quality; three datasets; all prostate) compared radiotherapy with other treatments [23,50,53]. Drummond et al. [23] reported statistically, but not clinically, significantly higher (worse) diarrhea scores in the radiotherapy group, Schwartz et al. [53] found statistically higher prevalence of diarrhea in the radiotherapy group, and Potosky et al. [50] found no statistical difference between groups.

Two gynecological papers from two datasets reported worse/higher prevalence of diarrhea in radiotherapy and non-radiotherapy groups [18,58]; statistical significance was reported in only one of these [58].

#### 3.7.7. Abdominal Pain/Painful Bowel Movements

Five papers (two of a high quality; three datasets; two prostate, one cervical) reported on abdominal pain or painful bowel movements [18,26,28,49,50]. The experience of symptoms ‘almost every day/some days’ among prostate survivors ranged from 10% at five years post-treatment [50] to 26% at six months [28]. Among gynecological survivors, prevalence was 27% for moderate or severe symptom distress and 48% for any distress [18].

One prostate paper reported a higher prevalence of symptoms at six months post-treatment compared with the baseline (statistical tests not reported) [28].

Two papers reported data from the same prostate dataset [49,50]. The prevalence of painful bowel movements declined from 26% to 14% at 12 and 24 months to 9% at 5 years.

One prostate paper comparing a radiotherapy and a non-cancer group reported higher prevalence in the radiotherapy group (statistical tests not reported) [26].

Two high-quality prostate papers from one dataset reported no statistical difference in the prevalence of pain in a radiotherapy group versus a surgery group [49,50]. Bergmark [18] found 48% of cervical cancer survivors in a radiotherapy group reported any distress from abdominal pain compared with 31% in a non-radiotherapy group. For those experiencing ‘moderate or much’ distress, the figures were 27% and 15% for radiotherapy and non-radiotherapy groups, respectively (statistical tests not reported).

#### 3.7.8. Painful Hemorrhoids

Three papers from one prostate dataset (two of a high quality) [28,49,50] reported a prevalence of painful hemorrhoids every day/almost every day ranging from 15% at six months post-treatment [28] to 20% at five years post-treatment [50]. In one of these studies, recall of painful hemorrhoids pre-diagnosis was reported by 10% of participants [28].

Two of these papers (both of a high quality) [49,50] also reported a statistically higher prevalence of symptoms in a radiotherapy compared with a surgery group.

#### 3.7.9. Rectal Wetness

Three papers [28,49,50] (two of a high quality; prostate; one dataset) reported on rectal wetness. Prevalence was 26% at 6 months [28], 21% at 24 months [49], and 18% almost every day at 5 years post-treatment [50]. In one of these studies, recall of rectal wetness pre-diagnosis was reported by 12% of participants [28].

Two of these papers (both of a high quality) compared radiotherapy and surgery groups [49,50] reporting the statistically higher prevalence of symptoms in the radiotherapy group at two years post-diagnosis but no difference at five years.

#### 3.7.10. Constipation

Drummond et al. [23] found statistically, but not clinically, significantly higher constipation scores (‘greater constipation problems’; EORTC QLQ-PR25) among prostate survivors treated with radiotherapy compared to those treated with surgery. A single study reported the lower prevalence of constipation distress in gynecological cancer survivors treated with radiotherapy than those treated with surgery (18%vs31%), but no formal statistical comparison was made [18].

#### 3.7.11. Mucous

Two papers (one of a high quality) [50,55] from different datasets reported on excessive rectal mucous discharge. Potosky et al. [50] found that the odds of excessive mucous among prostate cancer survivors were two-thirds lower in those treated with surgery when compared to those treated with radiotherapy; this was statistically significant. Steineck et al. [55] reported that a group of mucous-related symptoms were present in 16% of gynecological survivors treated with radiotherapy compared with no more than 5% of a non-cancer comparator group.

#### 3.7.12. Frequency

One paper [28] reported on frequent bowel movements (>3 movements per day), reporting that significantly more prostate survivors rarely or never experienced this at baseline (pre-diagnosis recall; 73%) as compared to 24 months (65%). The prevalence of frequent bowel movements ‘almost every day’ declined from 15% at 6 months to 12% at 12 months and 7% at 24 months (statistical tests not reported).

#### 3.7.13. Gas

One gynecological paper [55] identified a group of excessive gas-related symptoms present in 15% of cancer survivors compared with no more than 5% of a non-cancer comparator group.

## 4. Discussion

This systematic review aimed to identify and synthesize population-based data on the type, prevalence, and severity of bowel symptoms following pelvic radiotherapy, temporal trends in prevalence/severity, and associations with survivor characteristics. In total, 45 eligible papers reported on 12 distinct symptoms, though 7 were reported in fewer than 5 papers. The most commonly reported symptom was bowel function (often measured using EPIC-26), but even for this single symptom several different measures of function were reported (bowel function score, prevalence of a specified degree of patient-reported ‘bother’ or ‘distress’, or frequency of symptom occurrence).

Prevalence of problems varied by symptom, ranging from 1% for bleeding [31] to 59% (‘anal bleeding lasting more than 12 months at any time since start of treatment’) [36], and within symptoms (e.g., prevalence of bowel symptoms six months post-radiotherapy ranged from 4% to 16% across studies). However, in general, symptoms were reported more frequently in patients treated with radiotherapy for gynecological cancer as compared to those treated for prostate cancer, although the gynecological cancer studies were smaller so estimates would be less precise. In addition, there was a near universal pattern of higher prevalence or greater severity of symptoms in groups exposed to radiotherapy compared to those not exposed (non-cancer comparators or cancer patients who had undergone different treatments). This, in part, likely reflects the fact that the data collection tools used were intended to assess symptoms believed to be associated with exposure to pelvic radiotherapy. However, bowel symptoms are common in the general population, particularly among older groups [60], and (as shown in studies in this review and elsewhere) may also be reported by cancer survivors following treatments other than radiotherapy [50]. Therefore, while authors did not always report statistical testing, the consistent pattern of higher symptom prevalence in radiotherapy groups shown here provides further evidence of an excess of bowel symptoms following pelvic radiation. Having noted this, the evidence base would be strengthened if future studies included age- and sex-matched non-cancer comparator groups to better quantify the extent of this excess burden.

Bleeding and fecal incontinence are particularly problematic or distressing to cancer survivors [18,61] and potentially challenging for healthcare providers to manage; however, they were reported in relatively few papers (*n* = 7) and marked by low prevalence. Previous studies have reported higher prevalence of these particular bowel symptoms [61,62]. These differences may reflect continuing improvements in radiotherapy, leading to less damage (and less frequent symptoms) in more recently treated patient groups or selection bias in non-population-based studies. However, it has also been suggested that the methods commonly used in prospective studies to collect information on these problems routinely underestimate prevalence due to problems with symptom definition in survey instruments and because stigma and embarrassment leads survivors to under-report bowel symptoms [6,24]. Further, as has been observed elsewhere [63], it may be that those in poorest health, experiencing the highest symptom burden, are less likely to respond to questionnaires. Mortality is also likely to be higher among this group, which will lead to differential attrition in longitudinal studies with longer follow-up periods.

In the papers we reviewed, there was little systematic documentation of temporal trends in symptom prevalence post-treatment, and studies varied greatly in the time-points at which they assessed outcomes. Some papers suggested a peak in symptom prevalence around six months [28,33], and a reduction thereafter. Occasionally, however, there was a later increase. The lack of a clear, consistent pattern may reflect the small number of studies, heterogeneity in research methods, or differences in the underlying physiological processes. Different physiological processes can produce similar symptoms [1], and these might subsequently progress differently over time. A deeper understanding of temporal patterns may require the disaggregation of symptoms by underlying processes.

Previous, small, non-population-based studies have reported on survivor characteristics that may be risk or protective factors for bowel problems post-radiotherapy [64]. A large study within a prostate cancer trial population reported associations between late effects and both baseline health and acute symptoms [65]. There was a lack of these types of data from population-based sampling frames. The only data were related to variations in symptom risk or trends by ethnic group in USA studies, and the numbers of non-white individuals included were small. Greater understanding of whether particular survivor groups (e.g., older age, other medical conditions) are more likely to experience bowel problems post-radiotherapy could help to inform targeted support or follow-up. A similar approach has been used to identify patients most at risk of high morbidity following surgery for ovarian cancer [66], highlighting the potential for informing joint decision making with respect to treatment options.

We selected studies for review that used population-based sampling frames and patient-reported outcome measures. This was intended to increase the homogeneity and comparability of included studies. However, we found marked heterogeneity across multiple dimensions, including symptoms assessed, data collection tools used, comparator groups included, assessment time points, and cancer site treated (for studies of gynecological cancers). Additionally, radiotherapy regimens varied over time and institution. This heterogeneity precluded a statistical combination of study findings in a meta-analysis. However, our review highlights some specific gaps that might be addressed through future data collection and research to build a more consolidated evidence base.

Most of the 45 eligible papers from 19 datasets pertained to prostate cancer (39 papers, 17 datasets). There were fewer papers and only two datasets pertaining to gynecological cancer survivors (cervical cancer, endometrial cancer, or mixed gynecological cancers). There is a need for more population-based studies of gynecological cancers, for which the extent of damage to surrounding organs might be greater than for prostate cancer due to treatment usually involving irradiation of a larger area. There is also a need for more studies of less-researched cancers such as bladder cancer, the treatment of which, with radiotherapy, is also associated with bowel symptoms [67], but for which we identified no population-based studies.

The potential for learning from future studies would be improved through greater consistency in data collection methods across studies, including in the timing of data collection relative to treatment, the choice of outcomes, and data collection instrument. Regarding the latter, EPIC-26, though widely used, has possible shortcomings, including poor construct validity [68]. An alternative exists in the EORTC QLQ-PRT20 developed, with significant patient input, to assess bowel symptoms arising from radiotherapy treatment [69,70].

Our review did not consider the lived experiences of survivors with post-radiotherapy bowel problems. Evidence (albeit mainly anecdotal) suggests chronic bowel problems may have a substantial impact on peoples’ quality of life, but symptoms are under reported, under-recognized, and under-treated by health professionals [71,72,73]. There is, therefore, a need for research to better understand and document the impact on lives, strategies used to self-manage symptoms, and associated supportive care needs.

## 5. Conclusions

This review confirms that, following pelvic radiotherapy, cancer survivors may live, sometimes long-term, with a variety of chronic bowel symptoms. Worldwide, there are more than eight million survivors of prostate, endometrial, and cervical cancer [74], many of whom will have been previously treated with radiotherapy. Services and interventions to better support survivors experiencing bowel symptoms, coupled with more methodologically consistent research (including the use of consistent terminology) focusing on symptom prevalence, severity, risk, and impact in less-well-studied survivor populations, are urgently required.

## Figures and Tables

**Table 1:** Symptom prevalence at different timepoints.

Bowel Outcome	Author (Year of Publication)	Outcome Measurement Tool	Indicator	Cancer Site and Study Design	Prevalence (%) at Time Point Post-Treatment/Diagnosis (Years)										
					Baseline	0.5	1	2	3	4	5	6	9	12	15
Bowel symptoms	Baloch (2021) [15]	Study-specific	1 or more bowel syndromes	GC ^a^	-	-	-	-	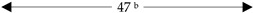						-
	Bandarage (2016) [17]	EPIC ^c^-26	Big bother	PL ^d^	-	-	4	5.1	-	-	-	-	-		-
	Gavin (2015) [27]	EORTC ^e^ QLQ ^f^-30 and PR ^g^25	Bowel problems	PC ^h^	-	-	-	22 ^i^ 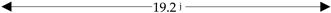 7.3 ^k^							
	Hamilton (2001) [28]	Study-specific	Moderate/big bother	PL	6.7	13.5	9.7	8.9	-	-	-	-	-	-	-
	Hoffman (2017) [30]	UCLA ^l^ PCI ^m^ and EPIC-26	Moderate/big bother	PC	-	-	-	-	-	-	-	-	-	-	15.6
	Hoffman (2020) [31]	EPIC-26	Moderate/big bother	PL	2	4	7	-	4	-	4	-	-	-	-
	Johnson (2004) [33]	UCLA PCI and EPIC-26	Bowel movement Problem moderate/big	PL	7.1	15.7	10	8.6	-	-	-	6.5	-	-	-
	Kyrdalen (2012) [34]	EPIC-50	Intestinal irritative symptoms	PC	-	-	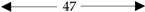			-	-		-	-	-
	nPotosky (2000) [49]	Study-specific	Urgency, frequency, or pain, moderate/big	PL	-	-	-	8.4	-	-	-	-	-	-	-
	nPotosky (2004) [50]	Study-specific	Urgency, frequency, or pain, moderate/big	PL	-	-	-	-	-	-	28.5	-	-		-
	Pryor (2021) [51]	EPIC-26	Moderate/big bother	PC	-	-	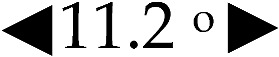	-	-	-	-	-	-		-
	Resnick (2013) [52]	Study-specific	Urgency, frequency, or pain, moderate/big	PL	-	-	-	7.9	-	-	5.8	-	-		-
	Schwartz (2002) [53]	Study-specific	Positive bother	PL	0.8	-	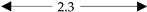			-	-	-	-		-
	Smith (2009) [54]	UCLA PCI	Bowel problems	PL	10.6 9^16^	-	-	-	14.5 12.5 ^p^	-	-	-	-		-
Urgency	Downing (2019) [22]	EPIC-26	Moderate/big problem	PC	-	-	-	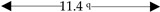			-	-	-		-
	Dunberger (2009) [25]	Study-specific	Urgency at least 1 × per week in past 6 months	GC	-	-	-	-	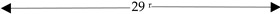						-
	Fossa (2022) [26]	EPIC-26	Moderate/big problem	PC	-	-	-	-	-	-	-	-	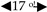	-	-
	Hamilton (2001) [28]	Study-specific	Urgent bowel movement everyday/almost every day	PL	2.2	7.9	4.8	3.5	-	-	-	-	-		-
	Hoffman (2020) [31]	EPIC-26	Urgency bother	PL	2	4	7	-	7	-	8	-	-		-
	Resnick (2013) [52]	Study-specific	Bowel urgency	PL	-	-	-	34	-	-	31.3	-	-		35.8
Bleeding	Dunberger (2009) [25]	Study-specific	Anal leakage of blood while awake Rectal bleeding	GC	-	-	-	-	7 ^q^ 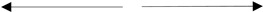 17 ^q^						-
	Fossa (2022) [26]	EPIC-26	Moderate/big problem	PC	-	-	-	-	-	-	-	-	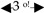	-	-
	Hoffman (2020) [31]	EPIC-26	Bloody stools	PL	1	1	1	-	2	-	0	-	-	-	-
	Potosky (2004) [50]	Study-specific	Bleeding with bowel movements	PL	-	-	-	-	-	-	13	-	-		-
Diarrhoea	Bergmark (2002) [18]	Study-specific	Loose stools distress much/moderate	GC	-	-	-	-	-	-	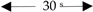		-	-	-
	nPotosky (2000) [49]	Study-specific	Every day/some days	PL	-	-	-	37.2	-	-	-	-			-
	nPotosky (2004) [50]	Study-specific	Every day/some days	PL	-	-	-	-	-	-	23.3	-			-
	Schwartz (2002) [53]	Study-specific	Loose stools	PL	6.2	-	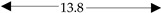			-	-	-			-
	Van de Poll-Franse (2012) [58]	EORTC QLQ-EN25 plusQLQ-30	Quite a bit/very much	GC	-	-	-	-	-	16	-	-	-	-	-
Incontinence	Dunberger (2010) [24]	Study-specific	Empty all stools into clothing	GC	-	-	-	-	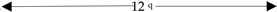						-
	Kyrdalen (2012) [34]	EPIC-50	Faecal leakage	PC	-	-	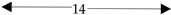			-	-	-			-
	Schwartz (2002) [53]	Study-specific	Faecal leakage more than a few drops	PL	1.5	-	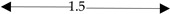			-	-	-			-
Abdominal pain	Bergmark (2002) [18]	Study-specific	Abdominal pain distress, much/moderate	GC	-	-	-	-	-	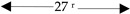		-		-	-
	Fossa (2022) [26]	EPIC-26	Moderate/big problem	PC	-	-	-	-	-	-	-	-	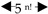		-
	Hamilton (2001) [28]	Study-specific	Pain with bowel movement, Every day/some days	PL	12	25.6	13.6	14	-	-	-	-	-	-	-
Painful haemorrhoids	Hamilton (2001) [28]	Study-specific	Every day/some days	PL	9.9	15.1	11.3	17.4	-	-	-	-			-
Rectal wetness	Hamilton (2001) [28]	Study-specific	Every day/some days	PL	12.4	26.4	22.3	19.6	-	-	-	-	-	-	-
Constipation	Bergmark (2002) [18]	Study-specific	Constipation distress much/moderate	GC	-	-	-	-	-	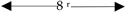		-	-	-	-
Mucous	Steineck (2017) [55]	Study-specific	Excessive mucous syndrome	GC	-	-	-	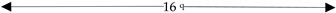							
Frequency	Hamilton (2001) [28]	Study-specific	>3 movements per day	PL	26.9	45.6	36.2	33.8	-	-	-	-			-
Gas	Steineck (2017) [55]	Study-specific	Excessive gas syndrome	GC	-	-	-	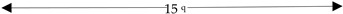							

Table 1 shows studies that present prevalence data for at least 1 timepoint defined relative to diagnosis/treatment or that provide the mean or median time since treatment/diagnosis or state the range of time since treatment/diagnosis. For studies that present prevalence data for defined time points, the prevalence is presented in the table for every time point reported. For studies that present a mean or median time since treatment/diagnosis, prevalence is included at the mean or median time rounded to the nearest whole year and arrows and vertical lines used to indicate the range rounded to the nearest whole years. For studies presenting only the range of time since treatment/diagnosis, the prevalence is presented centrally, with arrows and vertical lines indicating the range which is stated in the footnote for the table. One study per dataset is shown. When more than 1 study was reported on a single dataset, a single study was selected for inclusion in the table by prioritizing the most complete series of measurement points, then largest radiotherapy dataset, then earliest publication date. Where two studies from the same dataset present data for different time points, the data from both studies are presented. ^a^ Gynecological Cancer Cross Sectional Study, ^b^ no mean or median reported, ^c^ Expanded Prostate Cancer Index Composite, ^d^ Prostate Cancer Longitudinal Study, ^e^ European Organization for Research and Treatment of Cancer, ^f^ Quality of Life Questionnaire, ^g^ prostate cancer, ^h^ Prostate Cancer Cross Sectional Study, ^i^ External Bean Radiotherapy + Hormonal Therapy, ^j^ external beam radiotherapy, ^k^ brachytherapy, ^l^ University of California Los Angeles, ^m^ Prostate Cancer Index, ^n^ papers present non-overlapping data from the same dataset, ^o^ range not reported, ^p^ upper row = external beam radiotherapy and lower row = external beam radiotherapy + androgen depletion therapy, ^q^ data were collected 18–42 months post-diagnosis and no mean or median time point was reported, ^r^ data were collected 3–12 years post-diagnosis and no mean or median time point was reported, ^s^ data were collected 4–6 years post-diagnosis and no mean or median time point was reported, ^!^ datum read from graph.

**Table 2:** Summary of comparisons by symptom and dataset.

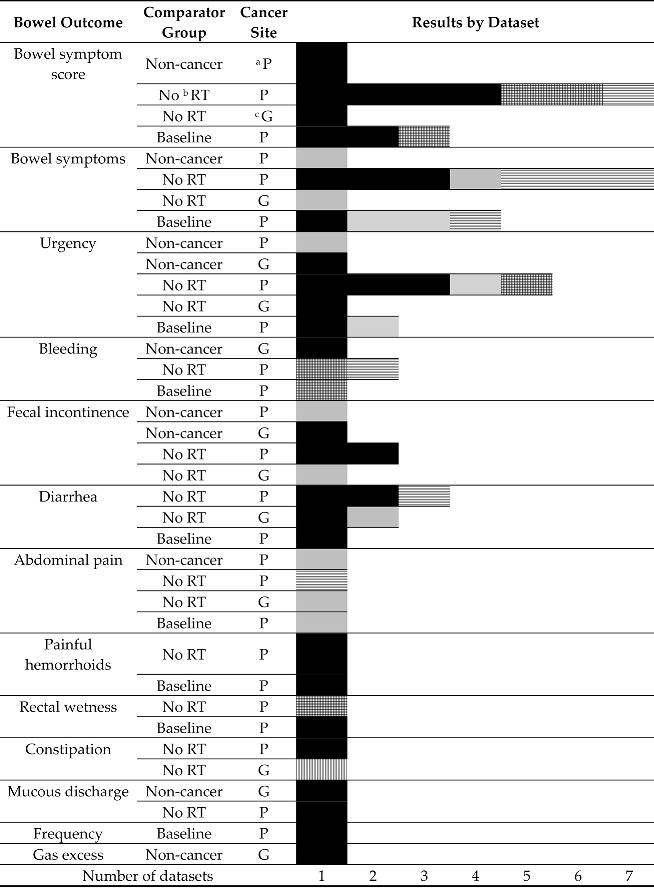

^a^ P = prostate, ^b^ RT = radiotherapy, ^c^ G = gynecological; 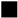 Statistically higher prevalence or greater severity in RT group. 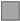 Higher prevalence or greater severity in RT group, statistical support not reported. 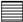 No statistical difference between groups. 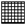 Mixed, some statistically different some not OR some papers present statistical support, some do not. All worse/higher in RT group. 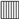 Lower prevalence in RT group, statistical support not reported.

## Data Availability

All data generated and/or analyzed during this review are included in this published article and/or associated supplementary material and/or are included in the published articles reviewed and referenced in this published article.

## Supplementary Materials

The following supporting information can be downloaded at: https://www.mdpi.com/article/10.3390/cancers15164037/s1, Table S1: Search strategies; Table S2: Study characteristics; Table S3: Population characteristics; Table S4: Outcome measures; Table S5: Quality appraisal; Table S6: Bowel outcome findings by symptom and paper.
