# Supplementary material for: A Systematic Review of Population-Based Studies of Chronic Bowel Symptoms in Cancer Survivors following Pelvic Radiotherapy
Source: Cancers (Basel). 2023 Aug 9;15(16):4037. doi: 10.3390/cancers15164037 (PMC10452492; doi:10.3390/cancers15164037)
Supplement: Supplementary file 1 [file cancers-15-04037-s001.zip › cancers-2527646-supplementary.pdf]

Contents

Supplementary Table S1. Search strategies 2
Supplementary Table S2. Study characteristics 9
Supplementary Table S3. Population characteristics 19
Supplementary Table S4. Outcome measures 30
Supplementary Table S5. Quality appraisal 31
Supplementary Table S6. Bowel outcome findings by symptom and paper 34

Supplementary Table S1. Search strategies

CINAHL

Search ID#	Search Terms	Search Options
S54	(S3 and S33 and S48) not S52	**Limiters** - English Language; Human; Publication Type: Journal Article<br>**Expanders** - Apply equivalent subjects<br>**Search modes** - Boolean/Phrase
S53	(S3 and S33 and S48) not S52	**Expanders** - Apply equivalent subjects<br>**Search modes** - Boolean/Phrase
S52	S49 OR S50 OR S51	**Expanders** - Apply equivalent subjects<br>**Search modes** - Boolean/Phrase
S51	TI clinical trial or randomi* controlled trial	**Expanders** - Apply equivalent subjects<br>**Search modes** - Boolean/Phrase
S50	(MH "Randomized Controlled Trials")	**Expanders** - Apply equivalent subjects<br>**Search modes** - Boolean/Phrase
S49	(MH "Clinical Trials")	**Expanders** - Apply equivalent subjects<br>**Search modes** - Boolean/Phrase
S48	S34 OR S35 OR S36 OR S37 OR S38 OR S39 OR S40 OR S41 OR S42 OR S43 OR S44 OR S45 OR S46 OR S47	**Expanders** - Apply equivalent subjects<br>**Search modes** - Boolean/Phrase
S47	diarr* or enterecolitis or tenesmus or flatulence or flatus or proctitis or proctopathy	**Expanders** - Apply equivalent subjects<br>**Search modes** - Boolean/Phrase
S46	(MH "Flatulence")	**Expanders** - Apply equivalent subjects<br>**Search modes** - Boolean/Phrase
S45	(MH "Diarrhea")	**Expanders** - Apply equivalent subjects<br>**Search modes** - Boolean/Phrase
S44	radiation enteropathy or pelvic radiation disease or radiation proctitis or radiation proctopathy	**Expanders** - Apply equivalent subjects<br>**Search modes** - Boolean/Phrase
S43	f#eces W3 incontinen* or or f#ecal W3 incontinen* or f#ecal W3 incontinen* or anal incontinen* or bowel W3 incontinen* or rectal W3 incontinen* or encopresis	**Expanders** - Apply equivalent subjects<br>**Search modes** - Boolean/Phrase
S42	(MH "Fecal Incontinence")	**Expanders** - Apply equivalent subjects<br>**Search modes** - Boolean/Phrase

S41	rectal adverse event or rectum adverse event or rectal bleed* or rectum bleed* or rectal complication* or rectum complication* or rectal dysfunction* or rectum dysfunction* or rectal effect* or rectum effect* or rectal function* or rectum function* or rectal injury or rectum injury or rectal mucosal damage or rectal problem* or rectum problem* or rectal symptom* or rectum symptom* or rectal toxic* or rectum toxic* or rectal hemorrhage or rectal haemorrhage or rectum haemorrhage or rectum hemorrh ...	**Expanders** - Apply equivalent subjects<br>**Search modes** - Boolean/Phrase
S40	intestin* injury or intestinal adverse event or intestin* bleed* or intestin* complication* or intestin* dysfunction* or intestin* effect* or intestinal frequency or intestin* function* or intestin* injury or intestin* mucosal damage or intestin* problem* or intestin* symptom* or intestin* toxic* or intestin* urgency	**Expanders** - Apply equivalent subjects<br>**Search modes** - Boolean/Phrase
S39	GI adverse event or GI bleed* or GI complication* or GI dysfunction* or GI effect* or GI frequency or GI function* or GI injury or GI mucosal damage or GI problem* or GI symptom* or GI toxic* or GI urgency	**Expanders** - Apply equivalent subjects<br>**Search modes** - Boolean/Phrase
S38	gastrointestinal disorder* or gastrointestinal hemorrhage or gastrointestinal haemorrhage or gastrointestinal adverse event or gastrointestinal bleed* or gastrointestinal complication* or gastrointestinal dysfunction* or gastrointestinal effect* or gastrointestinal frequency or gastrointestinal function* or gastrointestinal injury or gastrointestinal mucosal damage or gastrointestinal problem* or gastrointestinal symptom* or gastrointestinal toxic* or gastrointestinal urgency	**Expanders** - Apply equivalent subjects<br>**Search modes** - Boolean/Phrase
S37	(MH "Gastrointestinal Hemorrhage")	**Expanders** - Apply equivalent subjects<br>**Search modes** - Boolean/Phrase
S36	colorectal adverse event or colorectal bleed* or colorectal complication* or colorectal dysfunction* or colorectal effect* or colorectal frequency or colorectal function* or colorectal injury or colorectal mucosal damage or colorectal problem* or colorectal symptom* or colorectal toxic* or colorectal urgency	**Expanders** - Apply equivalent subjects<br>**Search modes** - Boolean/Phrase
S35	bowel adverse event or bowel bleed* or bowel complication* or bowel dysfunction* or bowel effect* or bowel frequency or bowel function* or bowel injury or bowel mucosal damage or bowel problem* or bowel symptom* or bowel toxic* or bowel urgency	**Expanders** - Apply equivalent subjects<br>**Search modes** - Boolean/Phrase
S34	anus injury or anal complaint or anal adverse event* or anal bleed* or anal complication* or anal dysfunction* or anal effect* or anal function* or anal injury anal problem* anal symptom* or anal toxic*	**Expanders** - Apply equivalent subjects<br>**Search modes** - Boolean/Phrase
S33	S4 OR S5 OR S6 OR S7 OR S8 OR S9 OR S10 OR S11 OR S12 OR S13 OR S14 OR S15 OR S16 OR S17 OR S18 OR S19 OR S20 OR S21 OR S22 OR S23 OR S24 OR S25 OR S26 OR S27 OR S28 OR S29 OR S30 OR S31 OR S32	**Expanders** - Apply equivalent subjects<br>**Search modes** - Boolean/Phrase
S32	vulva cancer* or vulva neoplasm* or vulva tumo* or vulva carcinoma* or cancer* of the vulva or vulval cancer* or vulval neoplasms or vulval tumo* or vulval carcinoma*	**Expanders** - Apply equivalent subjects<br>**Search modes** - Boolean/Phrase
S31	vaginal cancer* or vaginal neoplasm* or vaginal tumo* or vaginal carcinoma*	**Expanders** - Apply equivalent subjects

S30	vagina cancer* or vagina neoplasm* or vagina tumor* or vagina carcinoma* or cancer of the vagina	**Expanders** - Apply equivalent subjects<br>**Search modes** - Boolean/Phrase
S29	(MH "Vaginal Neoplasms")	**Expanders** - Apply equivalent subjects<br>**Search modes** - Boolean/Phrase
S28	uterus cancer* or uterus neoplasm* or uterus tumo* or uterus carcinoma* or cancer* of the uterus or uterine cancer or uterine neoplasm* or uterine tumo* or uterine carcinoma*	**Expanders** - Apply equivalent subjects<br>**Search modes** - Boolean/Phrase
S27	(MH "Uterine Neoplasms")	**Expanders** - Apply equivalent subjects<br>**Search modes** - Boolean/Phrase
S26	urinary tract cancer* or urinary tract neoplasm* or urinary tract tumo* or urinary tract carcinoma*or cancer* of the urinary tract	**Expanders** - Apply equivalent subjects<br>**Search modes** - Boolean/Phrase
S24	rectum cancer* or rectum neoplasm* or rectum tumo* or rectum carcinoma* or cancer* of the rectum or rectal cancer* or rectal neoplasm* or rectal tumo* or rectal carcinoma*	**Expanders** - Apply equivalent subjects<br>**Search modes** - Boolean/Phrase
S23	(MH "Rectal Neoplasms")	**Expanders** - Apply equivalent subjects<br>**Search modes** - Boolean/Phrase
S22	prostate cancer* or prostate neoplasm* or prostate tumo* or prostate carcinoma*or cancer of the prostate	**Expanders** - Apply equivalent subjects<br>**Search modes** - Boolean/Phrase
S21	(MH "Prostatic Neoplasms")	**Expanders** - Apply equivalent subjects<br>**Search modes** - Boolean/Phrase
S20	penis cancer* or penis neoplasm* or penis tumo* or penis carcinoma* or cancer* of the penis or penile cancer* or penile neoplasm* or penile tumo* or penile carcinoma*	**Expanders** - Apply equivalent subjects<br>**Search modes** - Boolean/Phrase
S18	pelvis cancer* or pelvis neoplasm* or pelvis tumo* or pelvis carcinoma* or pelvic cancer* or pelvic neoplasm* or pelvic tumo* or pelvic carcinoma* or cancer of the pelvis	**Expanders** - Apply equivalent subjects<br>**Search modes** - Boolean/Phrase
S17	(MH "Pelvic Neoplasms")	**Expanders** - Apply equivalent subjects<br>**Search modes** - Boolean/Phrase
S16	gynaecological cancer* or gynaecological neoplasm* or gynaecological tumo* or gynaecological carcinoma*	**Expanders** - Apply equivalent subjects<br>**Search modes** - Boolean/Phrase
S15	female genital tract cancer* or female genital tract neoplasm* or female genital tract tumo* or female genital tract carcinoma* or cancer* of the female genital tract	**Expanders** - Apply equivalent subjects<br>**Search modes** - Boolean/Phrase
S14	(MH "Genital Neoplasms, Female")	**Expanders** - Apply equivalent subjects<br>**Search modes** - Boolean/Phrase

S13	genitourinary cancer* or genitourinary neoplasm* or genitourinary tumo* or genitourinary carcinoma* or genitourinary tract cancer* or genitourinary tract neoplasms or genitourinary tract tumo* or genitourinary tract carcinoma* or cancer* of the genitourinary tract or urogenital cancer* or urogenital neoplasm* or urogenital tumo* or urogenital carcinoma* or urogenital tract cancer* or urogenital tract neoplasm* or urogenital tract tumo* or urogenital tract carcinoma* or cancer* of the urogenital ...	**Expanders** - Apply equivalent subjects<br>**Search modes** - Boolean/Phrase
S12	(MH "Urogenital Neoplasms")	**Expanders** - Apply equivalent subjects<br>**Search modes** - Boolean/Phrase
S11	endometrium cancer* or endometrium neoplasm* or endometrium tumo* or endometrium carcinoma* or cancer of the endometrium or endometrial cancer* or endometrial neoplasm* or endometrial tumo* or endometrial carcinoma*	**Expanders** - Apply equivalent subjects<br>**Search modes** - Boolean/Phrase
S10	(MH "Endometrial Neoplasms")	**Expanders** - Apply equivalent subjects
S9	uterine cervix cancer* or uterine cervix neoplasm* or uterine cervix tumo* or uterine cervix carcinoma* or cervical cancer* or cervical neoplasm* or cervical tumo* or cervical carcinoma* or cervix cancer* or cervix neoplasm* or cervix tumo* or cervix carcinoma* or cancer* of the cervix	**Expanders** - Apply equivalent subjects<br>**Search modes** - Boolean/Phrase
S8	(MH "Cervix Neoplasms")	**Expanders** - Apply equivalent subjects<br>**Search modes** - Boolean/Phrase
S7	bladder cancer* or bladder neoplasm* or bladder tumo* or bladder carcinoma* or cancer* of the bladder	**Expanders** - Apply equivalent subjects<br>**Search modes** - Boolean/Phrase
S6	(MH "Bladder Neoplasms")	**Expanders** - Apply equivalent subjects<br>**Search modes** - Boolean/Phrase
S5	anal cancer* or anal neoplasm* or anal tumo* or anal carcinoma* or anus cancer* or anus neoplasm* or anus tumo* or anus carcinoma* or cancer* of the anus	**Expanders** - Apply equivalent subjects<br>**Search modes** - Boolean/Phrase
S4	(MH "Anus Neoplasms")	**Expanders** - Apply equivalent subjects<br>**Search modes** - Boolean/Phrase
S3	S1 OR S2	**Expanders** - Apply equivalent subjects<br>**Search modes** - Boolean/Phrase
S2	chemoradiotherapy or irradiat* or radiation or radio treatment or radiotherapy	**Expanders** - Apply equivalent subjects<br>**Search modes** - Boolean/Phrase
S1	(MH "Radiotherapy")	**Expanders** - Apply equivalent subjects<br>**Search modes** - Boolean/Phrase

Embase

1.	cancer radiotherapy/
2.	(chemoradiotherapy or irradiat* or radiation or radio treatment or radiotherapy).mp.
3.	1 or 2
4.	anus cancer/
5.	(anal cancer* or anal neoplasm* or anal tumo* or anal carcinoma* or anus cancer* or anus neoplasm* or anus tumo* or anus carcinoma* or cancer* of the anus).mp.
6.	bladder cancer/
7.	(bladder cancer* or bladder neoplasm* or bladder tumo* or bladder carcinoma* or cancer* of the bladder).mp.
8.	uterine cervix cancer/
9.	(uterine cervix cancer* or uterine cervix neoplasm* or uterine cervix tumo* or uterine cervix carcinoma* or cervical cancer* or cervical neoplasm* or cervical tumo* or cervical carcinoma* or cervix cancer* or cervix neoplasm* or cervix tumo* or cervix carcinoma* or cancer* of the cervix).mp.
10.	endometrium cancer/
11.	(endometrium cancer* or endometrium neoplasm* or endometrium tumo* or endometrium carcinoma* or cancer of the endometrium or endometrial cancer* or endometrial neoplasm* or endometrial tumo* or endometrial carcinoma*).mp.
12.	urogenital tract cancer/
13.	(genitourinary cancer* or genitourinary neoplasm* or genitourinary tumo* or genitourinary carcinoma* or genitourinary tract cancer* or genitourinary tract neoplasms or genitourinary tract tumo* or genitourinary tract carcinoma* or cancer* of the genitourinary tract or urogenital cancer* or urogenital neoplasm* or urogenital tumo* or urogenital carcinoma* or urogenital tract cancer* or urogenital tract neoplasm* or urogenital tract tumo* or urogenital tract carcinoma* or cancer* of the urogenital tract).mp.
14.	female genital tract cancer/
15.	(female genital tract cancer* or female genital tract neoplasm* or female genital tract tumo* or female genital tract carcinoma* or cancer* of the female genital tract).mp.
16.	(gynaecological cancer* or gynaecological neoplasm* or gynaecological tumo* or gynaecological carcinoma*).mp.
17.	pelvis cancer/
18.	(pelvis cancer* or pelvis neoplasm* or pelvis tumo* or pelvis carcinoma* or pelvic cancer* or pelvic neoplasm* or pelvic tumo* or pelvic carcinoma* or cancer of the pelvis).mp.
19.	penis cancer/
20.	(penis cancer* or penis neoplasm* or penis tumo* or penis carcinoma* or cancer* of the penis or penile cancer* or penile neoplasm* or penile tumo* or penile carcinoma*).mp.
21.	prostate cancer/
22.	(prostate cancer* or prostate neoplasm* or prostate tumo* or prostate carcinoma* or cancer of the prostate).mp.
23.	rectum cancer/
24.	(rectum cancer* or rectum neoplasm* or rectum tumo* or rectum carcinoma* or cancer* of the rectum or rectal cancer* or rectal neoplasm* or rectal tumo* or rectal carcinoma*).mp.
25.	urinary tract cancer/
26.	(urinary tract cancer* or urinary tract neoplasm* or urinary tract tumo* or urinary tract carcinoma*or cancer* of the urinary tract).mp.
27.	uterus cancer/
28.	(uterus cancer* or uterus neoplasm* or uterus tumo* or uterus carcinoma* or cancer* of the uterus or uterine cancer or uterine neoplasm* or uterine tumo* or uterine carcinoma*).mp.
29.	vagina cancer/
30.	(vagina cancer* or vagina neoplasm* or vagina tumor* or vagina carcinoma* or cancer of the vagina).mp.
31.	(vaginal cancer* or vaginal neoplasm* or vaginal tumo* or vaginal carcinoma*).mp.
32.	vulva cancer/
33.	(vulva cancer* or vulva neoplasm* or vulva tumo* or vulva carcinoma* or cancer* of the vulva or vulval cancer* or vulval neoplasms or vulval tumo* or vulval carcinoma*).mp.
34.	4 or 5 or 6 or 7 or 8 or 9 or 10 or 11 or 12 or 13 or 14 or 15 or 16 or 17 or 18 or 19 or 20 or 21 or 22 or 23 or 24 or 25 or 26 or 27 or 28 or 29 or 30 or 31 or 32 or 33
35.	anus injury/
36.	(anus injury or anal complaint or anal adverse event* or anal bleed* or anal complication* or anal dysfunction* or anal effect* or anal function* or anal injury anal problem* anal symptom* or anal toxic*).mp.
37.	(bowel adverse event or bowel bleed* or bowel complication* or bowel dysfunction* or bowel effect* or bowel frequency or bowel function* or bowel injury or bowel mucosal damage or bowel problem* or bowel symptom* or bowel toxic* or bowel urgency).mp.
38.	(colorectal adverse event or colorectal bleed* or colorectal complication* or colorectal dysfunction* or colorectal effect* or colorectal frequency or colorectal function* or colorectal injury or colorectal mucosal damage or colorectal problem* or colorectal symptom* or colorectal toxic* or colorectal urgency).mp.
39.	gastrointestinal hemorrhage/
40.	gastrointestinal symptom/
41.	(gastrointestinal disorder* or gastrointestinal haemorrhage or gastrointestinal hemorrhage or gastrointestinal adverse event or gastrointestinal bleed* or gastrointestinal complication* or gastrointestinal dysfunction* or gastrointestinal effect* or gastrointestinal frequency or gastrointestinal function* or gastrointestinal injury or gastrointestinal mucosal damage or gastrointestinal problem* or gastrointestinal symptom* or gastrointestinal toxic* or gastrointestinal urgency).mp.
42.	(GI adverse event or GI bleed* or GI complication* or GI dysfunction* or GI effect* or GI frequency or GI function* or GI injury or GI mucosal damage or GI problem* or GI symptom* or GI toxic* or GI urgency).mp.
43.	intestinal bleeding/
44.	intestine injury/
45.	(intestin* injury or intestinal adverse event or intestin* bleed* or intestin* complication* or intestin* dysfunction* or intestin* effect* or intestinal frequency or intestin* function* or intestin* injury or intestin* mucosal damage or intestin* problem* or intestin* symptom* or intestin* toxic* or intestin* urgency).mp.
46.	rectum injury/
47.	rectum haemorrhage/
48.	(rectal adverse event or rectum adverse event or rectal bleed* or rectum bleed* or rectal complication* or rectum complication* or rectal dysfunction* or rectum dysfunction* or rectal effect* or rectum effect* or rectal function* or rectum function* or rectal injury or rectum injury or rectal mucosal damage or rectal problem* or rectum problem* or rectal symptom* or rectum symptom* or rectal toxic* or rectum toxic* or rectal hemorrhage or rectal haemorrhage or rectum haemorrhage or rectum hemorrhage or haematochezia).mp.
49.	feces incontinence/
50.	((((((((((((feces adj2 incontinen*) or faeces) adj2 incontinen*) or fecal) adj2 incontinen*) or faecal) adj2 incontinen*) or anal incontinen* or bowel) adj2 incontinen*) or rectal) adj2 incontinen*) or encopresis).mp.
51.	radiation enteropathy/
52.	(radiation enteropathy or pelvic radiation disease or radiation proctitis or radiation proctopathy).mp.
53.	diarrhea/
54.	enterecolitis/
55.	tenesmus/
56.	flatulence/
57.	(diarr* or enterecolitis or tenesmus or flatulence or flatus or proctitis or proctopathy).mp.
58.	35 or 36 or 37 or 38 or 39 or 40 or 41 or 42 or 43 or 44 or 45 or 46 or 47 or 48 or 49 or 50 or 51 or 52 or 53 or 54 or 55 or 56 or 57
59.	randomised controlled trial/
60.	clinical trial/
61.	(randomi* controlled trial or clinical trial).m_titl.
62.	59 or 60 or 61
63.	(3 and 34 and 58) not 62
64.	limit 63 to (human and english language and article and journal)

Medline

1.	radiotherapy/
2.	(chemoradiotherapy or irradiat* or radiation or radio treatment or radiotherapy).mp.
3.	1 or 2
4.	anus neoplasms/
5.	(anal cancer* or anal neoplasm* or anal tumo* or anal carcinoma* or anus cancer* or anus neoplasm* or anus tumo* or anus carcinoma* or cancer* of the anus).mp.
6.	urinary bladder neoplasms/
7.	(bladder cancer* or bladder neoplasm* or bladder tumo* or bladder carcinoma* or cancer* of the bladder).mp.
8.	uterine cervical neoplasms/
9.	(uterine cervix cancer* or uterine cervix neoplasm* or uterine cervix tumo* or uterine cervix carcinoma* or cervical cancer* or cervical neoplasm* or cervical tumo* or cervical carcinoma* or cervix cancer* or cervix neoplasm* or cervix tumo* or cervix carcinoma* or cancer* of the cervix).mp.
10.	endometrial neoplasms/
11.	(endometrium cancer* or endometrium neoplasm* or endometrium tumo* or endometrium carcinoma* or cancer of the endometrium or endometrial cancer* or endometrial neoplasm* or endometrial tumo* or endometrial carcinoma*).mp.
12.	urogenital neoplasms/
13.	(genitourinary cancer* or genitourinary neoplasm* or genitourinary tumo* or genitourinary carcinoma* or genitourinary tract cancer* or genitourinary tract neoplasms or genitourinary tract tumo* or genitourinary tract carcinoma* or cancer* of the genitourinary tract or urogenital cancer* or urogenital neoplasm* or urogenital tumo* or urogenital carcinoma* or urogenital tract cancer* or urogenital tract neoplasm* or urogenital tract tumo* or urogenital tract carcinoma* or cancer* of the urogenital tract).mp.
14.	Genital Neoplasms, Female/
15.	(female genital tract cancer* or female genital tract neoplasm* or female genital tract tumo* or female genital tract carcinoma* or cancer* of the female genital tract).mp.
16.	(gynaecological cancer* or gynaecological neoplasm* or gynaecological tumo* or gynaecological carcinoma*).mp.
17.	Pelvic Neoplasms/
18.	(pelvis cancer* or pelvis neoplasm* or pelvis tumo* or pelvis carcinoma* or pelvic cancer* or pelvic neoplasm* or pelvic tumo* or pelvic carcinoma* or cancer of the pelvis).mp.
19.	Penile Neoplasms/
20.	(penis cancer* or penis neoplasm* or penis tumo* or penis carcinoma* or cancer* of the penis or penile cancer* or penile neoplasm* or penile tumo* or penile carcinoma*).mp.
21.	Prostatic Neoplasms/
22.	(prostate cancer* or prostate neoplasm* or prostate tumo* or prostate carcinoma* or cancer of the prostate).mp.
23.	Rectal Neoplasms/
24.	(rectum cancer* or rectum neoplasm* or rectum tumo* or rectum carcinoma* or cancer* of the rectum or rectal cancer* or rectal neoplasm* or rectal tumo* or rectal carcinoma*).mp.
25.	Urologic Neoplasms/
26.	(urinary tract cancer* or urinary tract neoplasm* or urinary tract tumo* or urinary tract carcinoma*or cancer* of the urinary tract).mp.
27.	Uterine Neoplasms/
28.	(uterus cancer* or uterus neoplasm* or uterus tumo* or uterus carcinoma* or cancer* of the uterus or uterine cancer or uterine neoplasm* or uterine tumo* or uterine carcinoma*).mp.
29.	Vaginal Neoplasms/
30.	(vagina cancer* or vagina neoplasm* or vagina tumor* or vagina carcinoma* or cancer of the vagina).mp.
31.	(vaginal cancer* or vaginal neoplasm* or vaginal tumo* or vaginal carcinoma*).mp.
32.	Vulval Neoplasms/
33.	(vulva cancer* or vulva neoplasm* or vulva tumo* or vulva carcinoma* or cancer* of the vulva or vulval cancer* or vulval neoplasms or vulval tumo* or vulval carcinoma*).mp.
34.	4 or 5 or 6 or 7 or 8 or 9 or 10 or 11 or 12 or 13 or 14 or 15 or 16 or 17 or 18 or 19 or 20 or 21 or 22 or 23 or 24 or 25 or 26 or 27 or 28 or 29 or 30 or 31 or 32 or 33
35.	(anus injury or anal complaint or anal adverse event* or anal bleed* or anal complication* or anal dysfunction* or anal effect* or anal function* or anal injury anal problem* anal symptom* or anal toxic*).mp.
36.	(bowel adverse event or bowel bleed* or bowel complication* or bowel dysfunction* or bowel effect* or bowel frequency or bowel function* or bowel injury or bowel mucosal damage or bowel problem* or bowel symptom* or bowel toxic* or bowel urgency).mp.
37.	(colorectal adverse event or colorectal bleed* or colorectal complication* or colorectal dysfunction* or colorectal effect* or colorectal frequency or colorectal function* or colorectal injury or colorectal mucosal damage or colorectal problem* or colorectal symptom* or colorectal toxic* or colorectal urgency).mp.
38.	gastrointestinal hemorrhage/
39.	(gastrointestinal disorder* or gastrointestinal haemorrhage or gastrointestinal hemorrhage or gastrointestinal adverse event or gastrointestinal bleed* or gastrointestinal complication* or gastrointestinal dysfunction* or gastrointestinal effect* or gastrointestinal frequency or gastrointestinal function* or gastrointestinal injury or gastrointestinal mucosal damage or gastrointestinal problem* or gastrointestinal symptom* or gastrointestinal toxic* or gastrointestinal urgency).mp.
40.	(GI adverse event or GI bleed* or GI complication* or GI dysfunction* or GI effect* or GI frequency or GI function* or GI injury or GI mucosal damage or GI problem* or GI symptom* or GI toxic* or GI urgency).mp.
41.	(intestin* injury or intestinal adverse event or intestin* bleed* or intestin* complication* or intestin* dysfunction* or intestin* effect* or intestinal frequency or intestin* function* or intestin* injury or intestin* mucosal damage or intestin* problem* or intestin* symptom* or intestin* toxic* or intestin* urgency).mp.
42.	(rectal adverse event or rectum adverse event or rectal bleed* or rectum bleed* or rectal complication* or rectum complication* or rectal dysfunction* or rectum dysfunction* or rectal effect* or rectum effect* or rectal function* or rectum function* or rectal injury or rectum injury or rectal mucosal damage or rectal problem* or rectum problem* or rectal symptom* or rectum symptom* or rectal toxic* or rectum toxic* or rectal hemorrhage or rectal haemorrhage or rectum haemorrhage or rectum hemorrhage or haematochezia).mp.
43.	Fecal Incontinence/
44.	((((((((((((feces adj2 incontinen*) or faeces) adj2 incontinen*) or fecal) adj2 incontinen*) or faecal) adj2 incontinen*) or anal incontinen* or bowel) adj2 incontinen*) or rectal) adj2 incontinen*) or encopresis).mp.
45.	(radiation enteropathy or pelvic radiation disease or radiation proctitis or radiation proctopathy).mp.
46.	Diarrhea/
47.	Flatulence/
48.	(diarr* or enterecolitis or tenesmus or flatulence or flatus or proctitis or proctopathy).mp.
49.	36 or 37 or 38 or 39 or 40 or 41 or 42 or 43 or 44 or 45 or 46 or 47 or 48
50.	clinical trial/
51.	(randomi* controlled trial or clinical trial).m_titl.
52.	50 or 51
53.	(3 and 34 and 49) not 52
54.	limit 53 to (english language and humans and journal article)

PsychINFO

1.	radiation therapy/
2.	(chemoradiotherapy or irradiat* or radiation or radio treatment or radiotherapy).mp.
3.	1 or 2
4.	Neoplasms/
5.	(anal cancer* or anal neoplasm* or anal tumo* or anal carcinoma* or anus cancer* or anus neoplasm* or anus tumo* or anus carcinoma* or cancer* of the anus).mp.
6.	(bladder cancer* or bladder neoplasm* or bladder tumo* or bladder carcinoma* or cancer* of the bladder).mp.
7.	(uterine cervix cancer* or uterine cervix neoplasm* or uterine cervix tumo* or uterine cervix carcinoma* or cervical cancer* or cervical neoplasm* or cervical tumo* or cervical carcinoma* or cervix cancer* or cervix neoplasm* or cervix tumo* or cervix carcinoma* or cancer* of the cervix).mp.
8.	(endometrium cancer* or endometrium neoplasm* or endometrium tumo* or endometrium carcinoma* or cancer of the endometrium or endometrial cancer* or endometrial neoplasm* or endometrial tumo* or endometrial carcinoma*).mp.
9.	(genitourinary cancer* or genitourinary neoplasm* or genitourinary tumo* or genitourinary carcinoma* or genitourinary tract cancer* or genitourinary tract neoplasms or genitourinary tract tumo* or genitourinary tract carcinoma* or cancer* of the genitourinary tract or urogenital cancer* or urogenital neoplasm* or urogenital tumo* or urogenital carcinoma* or urogenital tract cancer* or urogenital tract neoplasm* or urogenital tract tumo* or urogenital tract carcinoma* or cancer* of the urogenital tract).mp.
10.	(female genital tract cancer* or female genital tract neoplasm* or female genital tract tumo* or female genital tract carcinoma* or cancer* of the female genital tract).mp.
11.	(gynaecological cancer* or gynaecological neoplasm* or gynaecological tumo* or gynaecological carcinoma*).mp.
12.	(pelvis cancer* or pelvis neoplasm* or pelvis tumo* or pelvis carcinoma* or pelvic cancer* or pelvic neoplasm* or pelvic tumo* or pelvic carcinoma* or cancer of the pelvis).mp.
13.	(penis cancer* or penis neoplasm* or penis tumo* or penis carcinoma* or cancer* of the penis or penile cancer* or penile neoplasm* or penile tumo* or penile carcinoma*).mp.
14.	(prostate cancer* or prostate neoplasm* or prostate tumo* or prostate carcinoma* or cancer of the prostate).mp.
15.	(rectum cancer* or rectum neoplasm* or rectum tumo* or rectum carcinoma* or cancer* of the rectum or rectal cancer* or rectal neoplasm* or rectal tumo* or rectal carcinoma*).mp.
16.	(urinary tract cancer* or urinary tract neoplasm* or urinary tract tumo* or urinary tract carcinoma*or cancer* of the urinary tract).mp.
17.	(uterus cancer* or uterus neoplasm* or uterus tumo* or uterus carcinoma* or cancer* of the uterus or uterine cancer or uterine neoplasm* or uterine tumo* or uterine carcinoma*).mp.
18.	(vagina cancer* or vagina neoplasm* or vagina tumor* or vagina carcinoma* or cancer of the vagina).mp.
19.	(vaginal cancer* or vaginal neoplasm* or vaginal tumo* or vaginal carcinoma*).mp.
20.	(vulva cancer* or vulva neoplasm* or vulva tumo* or vulva carcinoma* or cancer* of the vulva or vulval cancer* or vulval neoplasms or vulval tumo* or vulval carcinoma*).mp.
21.	4 or 5 or 6 or 7 or 8 or 9 or 10 or 11 or 12 or 13 or 14 or 15 or 16 or 17 or 18 or 19 or 20
22.	(anus injury or anal complaint or anal adverse event* or anal bleed* or anal complication* or anal dysfunction* or anal effect* or anal function* or anal injury anal problem* anal symptom* or anal toxic*).mp.
23.	(bowel adverse event or bowel bleed* or bowel complication* or bowel dysfunction* or bowel effect* or bowel frequency or bowel function* or bowel injury or bowel mucosal damage or bowel problem* or bowel symptom* or bowel toxic* or bowel urgency).mp.
24.	(colorectal adverse event or colorectal bleed* or colorectal complication* or colorectal dysfunction* or colorectal effect* or colorectal frequency or colorectal function* or colorectal injury or colorectal mucosal damage or colorectal problem* or colorectal symptom* or colorectal toxic* or colorectal urgency).mp.
25.	gastrointestinal disorders/
26.	(gastrointestinal disorder* or gastrointestinal haemorrhage or gastrointestinal hemorrhage or gastrointestinal adverse event or gastrointestinal bleed* or gastrointestinal complication* or gastrointestinal dysfunction* or gastrointestinal effect* or gastrointestinal frequency or gastrointestinal function* or gastrointestinal injury or gastrointestinal mucosal damage or gastrointestinal problem* or gastrointestinal symptom* or gastrointestinal toxic* or gastrointestinal urgency).mp.
27.	(GI adverse event or GI bleed* or GI complication* or GI dysfunction* or GI effect* or GI frequency or GI function* or GI injury or GI mucosal damage or GI problem* or GI symptom* or GI toxic* or GI urgency).mp.
28.	(intestin* injury or intestinal adverse event or intestin* bleed* or intestin* complication* or intestin* dysfunction* or intestin* effect* or intestinal frequency or intestin* function* or intestin* injury or intestin* mucosa damage or intestin* problem* or intestin* symptom* or intestin* toxic* or intestin* urgency).mp.
29.	(rectal adverse event or rectum adverse event or rectal bleed* or rectum bleed* or rectal complication* or rectum complication* or rectal dysfunction* or rectum dysfunction* or rectal effect* or rectum effect* or rectal function* or rectum function* or rectal injury or rectum injury or rectal mucosal damage or rectal problem* or rectum problem* or rectal symptom* or rectum symptom* or rectal toxic* or rectum toxic* or rectal hemorrhage or rectal haemorrhage or rectum haemorrhage or rectum hemorrhage or haematochezia).mp.
30.	Fecal Incontinence/
31.	((((((((((((feces adj2 incontinen*) or faeces) adj2 incontinen*) or fecal) adj2 incontinen*) or faecal) adj2 incontinen*) or anal incontinen* or bowel) adj2 incontinen*) or rectal) adj2 incontinen*) or encopresis).mp.
32.	(radiation enteropathy or pelvic radiation disease or radiation proctitis or radiation proctopathy).mp.
33.	Diarrhea/
34.	(diarr* or enterecolitis or tenesmus or flatulence or flatus or proctitis or proctopathy).mp.
35.	22 or 23 or 24 or 25 or 26 or 27 or 28 or 29 or 30 or 31 or 32 or 33 or 34
36.	clinical trial/
37.	(randomi* controlled trial or clinical trial).m_titl.
38.	36 or 37
39.	(3 and 21 and 35) not 38
40.	limit 39 to (human and english language and "0110 peer-reviewed journal" and journal article)

Supplementary Table S2. Study characteristics

Country in which patients were recruited	First Author (year of publication)	Study Design	Control / Comparator Group	Measurement Time Points	Bowel Symptom Measurement Tool	Bowel Symptoms Reported	Quality Appraisal Score [1]
Australia and New Zealand	Pryor (2021)	Cross sectional	RT[2] groups<br>HRT[3] v CRT[4]	Since start of treatment<br>Days<br>Median (IQR[5])<br>407 (371-449)	EPIC[6]-26	Bowel function score, bowel bother	6/14
Australia	Bandarage (2016)	Longitudinal	None	Since diagnosis<br>T[7]1=12 months<br>T2=24 months	EPIC-26	Bowel bother	9/14
Australia	Mazariego (2020)	Longitudinal	Non-cancer	Since diagnosis<br>T1=Baseline (recall of pre-diagnosis)<br>T2=6 months<br>T3=1year<br>T4=2 years<br>T5=3years<br>T6=5 years<br>T7=10 years<br>T8=15 years	Subset of items common to both UCLA-PCI[8] and EPIC-26	Moderate or severe bowel problems.<br>Bowel bother score	15/20
Australia	Ong (2022)	Cross sectional	RT groups<br>EBRT[9] v EBRT+BT[10]	Since treatment<br>12 months	EPIC-26	Bowel function score	6/14
Australia	Smith (2009)	Longitudinal	Non-cancer	Since diagnosis<br>T1=baseline recall within 12 months of diagnosis<br>T2= 1 year<br>T3=3 years	UCLA-PCI	Bowel function, bowel bother	15/20

Country in which patients were recruited	First Author (year of publication)	Study Design	Control / Comparator Group	Measurement Time Points	Bowel Symptom Measurement Tool	Bowel Symptoms Reported	Quality Appraisal Score [1]
Denmark	Nguyen-Nielson (2019)	Longitudinal	Other-Tx[11] (AS[12])	Since diagnosis T1=diagnosis T2=1 year T2=3 years	EPIC-26	Bowel domain score	13/20
Finland	Lehto (2017)	Cross sectional	Other-Tx (RP[13])	Since diagnosis 5 years	Study specific[14]	Anal bleeding	9/20
Ireland and Northern Ireland	Drummond (2015)	Cross sectional	Other-Tx (RP)	Since diagnosis Years Total sample <5 48%, 5-10 32%, 10+ 20%, EBRT+ADT <5 65%, 5-10 27%, 10+ 9%, EBRT <5 43%, 5-10 37%, 10+ 21% BT <5 70%, 5-1- 27%, 10+ 3%	EORTC[15] QLQ-C[16]30 and EORTC QLQ-PR[17]25	Diarrhoea, constipation, bowel symptoms	12/20
Ireland and Northern Ireland	Gavin (2015)	Cross sectional	Other-Tx (RP, AS/WW[18])	Since diagnosis Years 2-4.9 8.2%, 5-9.9 32.1%, 10+ 21.3%	EORTC QLQ-C30 and EORTC QLQ-PR25,	Bowel problems ever and current (diarrhoea, constipation)	11/20
Netherlands	Mols (2008)	Cross sectional	Non-cancer	Since diagnosis, years, 5-7 62% 8-10 38%	EPIC-26	Bowel functioning, bowel bother, bowel summary	12/20

Country in which patients were recruited	First Author (year of publication)	Study Design	Control / Comparator Group	Measurement Time Points	Bowel Symptom Measurement Tool	Bowel Symptoms Reported	Quality Appraisal Score [1]
Netherlands	Van de Poll-Franse (2012)	Cross sectional	Other-Tx (surgery)	Since diagnosis, Years Mean (SD[19]) 4.4 (2.4)	EORTC QLQ-EN[20]24	Gastrointestinal symptoms scale, diarrhoea scale	10/20
Norway	Fossa (2022)	Cross sectional	Non-cancer	Since treatment Years Mean 9	EPIC-26	Bowel function score and prevalence of moderate or big problems with; frequency, urgency, bleeding, incontinence, pain and overall bowel function	5/20
Norway	Kyrdalen (2012)	Cross sectional	Other-Tx (No treatment, RP)	Since start of treatment Months Median (range), 23 (12-32)	selected items from EPIC-50	Intestinal irritative symptoms (1 or more of defecation 3+ daily, at least half the time diarrhoea, blood or mucus in stool, painful defecation. Faecal leakage	10/20
Sweden	Baloch (2021)	Cross sectional	None	Since treatment Years 3-15	Study specific	Number of syndromes (1, 2 or 3), prevalence of syndromes (urgency, faecal leakage, bleeding)	6/14

Country in which patients were recruited	First Author (year of publication)	Study Design	Control / Comparator Group	Measurement Time Points	Bowel Symptom Measurement Tool	Bowel Symptoms Reported	Quality Appraisal Score [1]
Sweden	Bergmark (2002)	Cross sectional	Non-cancer	Since diagnosis, Years 4-5	Study specific	Symptom related distress associated with abdominal pain, constipation, bowel dysfunction, loose stool, urgency, leakage.	7/20
Sweden	Carlsson (2015)	Cross sectional	Non-cancer	Since diagnosis Median (IQR) 12 (11-13) years	Study specific	Bowel dysfunction	12/20
Sweden	Dunberger (2009)	Cross sectional	Non-cancer	Since treatment Years 1-13	Study specific	32 reported symptoms including urgency, empty all stools into clothing without warning, anal leakage of blood while awake, rectal bleeding	12/20
Sweden	Dunberger (2010)	Cross sectional	Non-cancer	Since treatment Months Mean (SD) With faecal incontinence 86.2 (38) No faecal incontinence 86 (40.8)	Study specific	Emptied all stools into clothing without warning at least occasionally (faecal incontinence)	13/20

Country in which patients were recruited	First Author (year of publication)	Study Design	Control / Comparator Group	Measurement Time Points	Bowel Symptom Measurement Tool	Bowel Symptoms Reported	Quality Appraisal Score [1]
Sweden	Steineck (2017)	Cross sectional	Non-cancer	Since treatment Years $\leq$ 15	Study specific	5 syndromes; urgency, leakage, gas, mucus, blood	12/20
UK	Downing (2019)	Cross sectional	Other-Tx (RP)	Since diagnosis Months 18-42	EPIC-26	Bowel function score, urgency, frequency, incontinence, bloody stools, rectal pain, overall bowel	6/20
UK	Mason (2021)	Longitudinal (pre and post-additional treatment)	Other-Tx (RP, ADT[21])	Since diagnosis Months T1=18-42 T2=12 after T1	EPIC-26	Bowel function	13/20
UK (England)	Nossiter (2020)	Cross sectional	RT groups CRT vs HRT	Since treatment, Months 6-12 11.4% 12-18 55.8% 18-24 21.1% 24+ 11.7%	EPIC-26	Bowel domain score	6/14
UK (England)	Parry (2020)	Cross sectional	RT groups RT with vs without lymph node inclusion	Since treatment Months Median (IQR) PO[22] IMRT[23] 16.2 (13.8-22.1) PPLN[24] IMRT 15.1 (13.0-19.1)	EPIC-26	Bowel function domain score	6/14

Country in which patients were recruited	First Author (year of publication)	Study Design	Control / Comparator Group	Measurement Time Points	Bowel Symptom Measurement Tool	Bowel Symptoms Reported	Quality Appraisal Score [1]
UK (England)	Parry (2020)	Cross sectional	RT groups<br><br>EBRT v EBRT-BT boost	Since treatment Months<br>6-11 11.3%<br>12-18, 55.8%	EPIC-26	Bowel function domain score	7/14
UK (England)	Parry (2021)	Cross sectional	RT groups<br>EBRT v HDR[25] or LDR[26] BT boost	Since diagnosis Months<br>$\geq$ 18	EPIC-26	Bowel function domain score	5/14
USA	Barocas (2017)	Longitudinal	Other Tx (AS)	Since enrolment (Enrolled within 6 months of diagnosis)<br>T1= 6 months<br>T2=12 months<br>T3=36 months	EPIC-26	Bowel function domain score, bowel function bother, bloody stools, bowel urgency	14/20
USA	Chen (2017)	Longitudinal	Other Tx (AS)	Since treatment<br>T1=pre-treatment<br>T2=3 months<br>T3=12 months<br>T4=24 months	PCSI	bowel problems	14/20
USA	De (2022)	Longitudinal	Other-Tx (RP)	Since diagnosis<br>T1=diagnosis<br>T2=6 months<br>T3=1 year<br>T3=3 years<br>T4-5 years	EPIC-26	Bowel function domain score, bowel function bother, bloody stools, bowel urgency	13/20

Country in which patients were recruited	First Author (year of publication)	Study Design	Control / Comparator Group	Measurement Time Points	Bowel Symptom Measurement Tool	Bowel Symptoms Reported	Quality Appraisal Score [1]
USA	Hamilton (2001)	Longitudinal	None	Since diagnosis T1=6 months T2=12 months T3=24 months	Study specific	> 3 movements per day, pain with bowel movement, urgency, rectal wetness, painful haemorrhoids, How big a problem is bowel function	10/14
USA	Heulster (2020)	Longitudinal	Other-Tx (RP)	Since diagnosis T1<6 months T2= 6 months T3=12 months T4=36 months T5=60 months	EPIC-26	Bowel function score / bowel irritative domain score	13/20
USA	Hoffman (2003)	Cross Sectional	Other-Tx (No treatment, ADT, RP)	Since diagnosis, Months 24	Not specified	Urgency	14/20
USA	Hoffman (2017)	Cross sectional	Other-Tx (RP, WW)	Since diagnosis Years 15	UCLA PCI and EPIC-26	Bowel bother	11/20
USA	Hoffman (2020)	Longitudinal	Other-Tx (RP and AS)	Since diagnosis T1=baseline T2=6 months T3=1 year T4=3 years T5=5 years	EPIC-26	EPIC-26 bowel domain score, bowel function bother, bloody stools, bowel urgency	9/20

Country in which patients were recruited	First Author (year of publication)	Study Design	Control / Comparator Group	Measurement Time Points	Bowel Symptom Measurement Tool	Bowel Symptoms Reported	Quality Appraisal Score [1]
USA	Johnson (2004)	Longitudinal	None	Since diagnosis T1=6 months T2=12 months T3=24 months T5=60 months	Study specific	Urgency How big a problem	14/14
USA	Lee (2018)	Longitudinal	RT groups EBRT v EBRT+LDR-BT	Since diagnosis T1=diagnosis T2=6 months T3=1 year T3=3 years	EPIC-26	Bowel function score - problem with bowel function; problem with bowel urgency	9/14
USA	Moon (2019)	Longitudinal	Other-Tx (AS)	T1=1-2 weeks post-diagnosis<br><br>Since treatment T2=3 months T3=12 months T4=24 months	PCSI	Bowel Problems score	9/20
USA	O'Neil (2018)	Longitudinal	RT groups<br><br>IMRT vs mid 1990s EBRT	Since diagnosis T1=diagnosis T2=6 months T3=12 months	EPIC-26 and UCLA PCI	Bowel function	13/14
USA	Pasalic (2021)	Longitudinal	RT groups EBRT v EBRT+LDR BT	Since enrolment T1=6 months T2-1 year T2=3 years T3=5 years	EPIC-26	Bowel function domain score, bowel function bother, bloody stools, bowel urgency	8/14

Country in which patients were recruited	First Author (year of publication)	Study Design	Control / Comparator Group	Measurement Time Points	Bowel Symptom Measurement Tool	Bowel Symptoms Reported	Quality Appraisal Score [1]
USA	Potosky (2000)	Longitudinal	Other-Tx (RP)	T1=enrolment (within 6 months of diagnosis)<br>T2=2 years since T1	Study specific	Bowel symptoms, diarrhoea, painful bowel movements, bowel urgency, wetness in rectal area, painful haemorrhoids, bothered by frequent movements	15/20
USA	Potosky (2004)	Longitudinal	Other-Tx (RP)	T1= within 6 months of diagnosis<br>Since T1<br>T2=2 years<br>T3=5 years	Study specific	Bowel symptoms, diarrhoea, painful bowel movements, bowel urgency, wetness in rectal area, painful haemorrhoids, bothered by frequent movements	14/20
USA	Resnick (2013)	Longitudinal	Other-Tx (RP)	Since diagnosis<br>T1 within 12 months<br>T2=2 years<br>T3=5 years<br>T4=15 years	Study specific	Bowel urgency, bothered by frequent bowel movements	13/20

Country in which patients were recruited	First Author (year of publication)	Study Design	Control / Comparator Group	Measurement Time Points	Bowel Symptom Measurement Tool	Bowel Symptoms Reported	Quality Appraisal Score [1]
USA	Schwartz (2002)	Longitudinal	Other-Tx (RP)	Since treatment<br>T1=mean 9 months<br>T2=mean 2 years	Study specific	Loose stools, stool leakage, leakage greater than few drops, use of pads, positive bother	16/20
USA	Tyson (2016)	Longitudinal	Other-Tx (AS or RP)	Since diagnosis<br>T1=diagnosis<br>T2=6 months<br>T3-12 months	EPIC-26	Bowel function domain score	13/20
USA	Tyson (2018)	Longitudinal	Other-Tx (RP)	Since diagnosis<br>T1=diagnosis<br>T2=6 months<br>T3-12 months<br>T4=36 months	EPIC-26	Bowel function domain score	12/20
USA	Wallis (2022)	Longitudinal	RT groups<br>RT to prostate only v prostate and pelvis	T1=within 6 months of diagnosis<br>T2=6 months<br>T3=1 year<br>T4=3 years<br>T5=5 years<br>From T1	EPIC-26	Bowel function score	8/14

[1] Score out of a possible total of 14 for a study without a non-radiotherapy or non-cancer comparator group and a possible total of 20 for a study with such a comparator group, [2] Radiotherapy, [3]Hypofractionated Radiotherapy, [4]Conventional Radiotherapy, [5]Inter Quartile Range,[6] Expanded Prostate Cancer Index Composite, [7]Timepoint (of outcome measurement), [8]University of California Los Angeles Prostate Cancer Index, [9]External Beam Radiotherapy, [10]Brachytherapy, [11]Treatments, [12]Active Surveillance, [13]Radical Prostatectomy, [14] Study specific includes tools created for the study and those adapted or derived from existing tools, [15]European Organisation for Research and Treatment of Cancer, [16]Quality of Life Questionnaire-Core, [17]Quality of Life Questionnaire-Prostate Cancer, [18]Watchful Waiting, [19]Standard Deviation, [20]Quality of Life Questionnaire Endometrial Cancer, [21]Androgen Deprivation Therapy, [22]Pelvis Only, [23]Intensity Modulated Radiotherapy, [24]Pelvis Plus Lymph Node, [25]High Dose Rate, [26]Low Dose Rate

Supplementary Table S3. Population characteristics

Country of recruitment	Source of patients	Year of diagnosis / treatment	First Author (year of publication)	Sample size [1]	Cancer site	Extent of disease [2]	Age (years) [3]	Race / ethnicity [4]
Australia	New South Wales Central Cancer Registry	Diagnosed 2000-2002	Smith (2009)	1,995	Prostate	Gleason Score<br>EBRT[5]<br>2-6 47.1%<br>7 45.5%<br>8-10 7.4%<br><br>EBRT/ADT[8]<br>2-6 29.5%<br>7 42.8%<br>8-10 27.7%	At diagnosis<br>Mean (CI[6])<br>EBRT<br>63.9(63, 64.7)<br><br>EBRT/ADT<br>63.3(62.5, 64)	NR[7]
Australia	New South Wales Central Cancer Registry	Diagnosed 2000-2002	Mazariego (2020)	1,642	Prostate	Localised	NR	NR
Australia	Victorian Prostate Cancer Registry	Diagnosed 2009-2014	Bandarage (2016)	1,825 (at 24-month follow-up)	Prostate	Localised	Median (IQR[9])<br>66, (60, 71.7)	NR
Australia	Victorian Prostate Cancer Registry	Diagnosed 2015-2020	Ong (2022)	1,555	Prostate	Low risk 1.8%, intermediate risk 45.7%, high risk 40.8%	At treatment<br>Mean (SD[10])<br>72. 8 (6.5)	NR
Australia and New Zealand	Australia and New Zealand Prostate Cancer Outcomes Registry (PCOR-ANZ)	Treated 2016-2019	Pryor (2021)	3531	Prostate	Low risk 2.6%<br>Intermediate risk 45.7%<br>high/very high risk 44.5%<br>Regional (cN1) 7.1%	At start of treatment,<br>Median (IQR),<br>73.1 (68.2, 77.3)	NR
Denmark	Danish Prostate Cancer Registry	Diagnosed 2010-2016	Nguyen-Nielsen (2019)	15,465	Prostate	Gleason Score<br>≤6 28.3%	Median (IQR)<br>69 (64, 74)	NR

Country of recruitment	Source of patients	Year of diagnosis / treatment	First Author (year of publication)	Sample size [1]	Cancer site	Extent of disease [2]	Age (years) [3]	Race / ethnicity [4]
						7 42.6%<br>≥8 29.1%		
Finland	Finnish Cancer Registry	Diagnosed 2004	Lehto (2017)	1,239	Prostate	Gleason score<br>≤4 10%<br>5-6 40%<br>7 26%<br>8-10 13%	≤64 20%<br>65-74 41%<br>75-84 34%<br>85+ 5%	Homogeneous
Ireland and Northern Ireland	National Cancer Registry Ireland & Northern Ireland Cancer Registry	Diagnosed 1995-2010	Drummond (2015)	3,348	Prostate	Gleason score<br>≤6 6%,<br>7-8 65%,<br>8-10 19%	<60 24%<br>60-69 49%<br>≥70 27%	NR
Ireland and Northern Ireland	National Cancer Registry Ireland & Northern Ireland Cancer Registry	Diagnosed 1995-2010	Gavin (2015)	3,348	Prostate	Gleason score<br>2-4 6.9%<br>5-7 51.6%<br>8-10 12.6%	Mean (SD)<br>64.9 (7.6)	NR
Netherlands	Eindhoven Cancer Registry	Diagnosed 1994-1998	Mols (2008)	780	Prostate	Clinical stage at diagnosis (TNM[11] clinical classification)<br>RT[12] group<br>I 27%<br>II 65%<br>III 5%	RT group<br>Mean 76	NR
Netherlands	Eindhoven Cancer Registry	Diagnosed 1999-2007	Van de Poll-Franse (2012)	742	Endometrial	FIGO[13] stage at diagnosis<br>IA 15.8%A<br>IB 50.5%<br>IC 26.2%<br>II 3.8%<br>IIB 3.8	Mean (SD)<br>66.7 (8.5)	NR

Country of recruitment	Source of patients	Year of diagnosis / treatment	First Author (year of publication)	Sample size [1]	Cancer site	Extent of disease [2]	Age (years) [3]	Race / ethnicity [4]
Norway	Cancer Registry of Norway	Diagnosed 2004-2015	Fossa (2022)	4,387	Prostate	NR	Mean (SD) Cancer group 74.5 (4.3), Control group 73.4 (4.9)	NR
Norway	Cancer Registry of Norway and Norwegian Prescription Database	Diagnosed 2004	Kyrdalen (2012)	771	Prostate	Slightly modified D'Amico risk assessment) High risk 25% Intermediate risk 33% Low risk 43%	Median 66, range 45-75	NR
Sweden	Swedish National Prostate Cancer Registry	Diagnosed 1997-2002	Carlsson (2015):	5,944	Prostate	Low risk 55.5%, Intermediate risk 40.4% High risk 4.1%.	At diagnosis Median (IQR) 63, (59-67)	NR
Sweden	All (7) Swedish departments of gynaecological oncology	Diagnosed 1991-1992	Bergmark (2002)	256 (cancer survivors) 158 (non-cancer controls)	Cervical	Early (FIGO stage IB-IIA)	Mean ([14]SE) 51 (0.77)	NR
Sweden	Karolinska University Hospital, Stockholm and Jubileumskliniken, Sahlgren University Hospital, Gothenburg	Treated 1991-2003	Dunberger (2009)	616 (cancer survivors) 344 (non-cancer controls)	Gynaecological (Endometrial 59%, Cervical 23%, Ovarian 7%, Uterine 5%, Vaginal 2%, Fallopian	NR	Survivors 28-38 11% 39-49 8% 50-60 19% 61-71 41% 72-79 29% Controls	NR

Country of recruitment	Source of patients	Year of diagnosis / treatment	First Author (year of publication)	Sample size [1]	Cancer site	Extent of disease [2]	Age (years) [3]	Race / ethnicity [4]
					tube 2%, Vulvar 1%)		28-38 7%<br>39-49 23%<br>50-60 25%<br>61-71 25%<br>72-79 19%	
Sweden	Karolinska University Hospital, Stockholm and Jubileumskliniken, Sahlgren University Hospital, Gothenburg	Treated 1991-2003	Dunberger (2010)	616 (cancer survivors)<br>344 (non-cancer controls)	Gynaecological	NR	Mean<br><br>Survivors reporting faecal incontinence 65.8<br><br>Survivors not reporting faecal incontinence 64.1<br><br>Controls 58	NR
Sweden	Radiumhemmet, Karolinska University Hospital in Stockholm and Jubileumskliniken, Sahlgrenska University Hospital in Gothenburg	Treated 1991-2003	Steineck (2017)	650	Gynaecological	NR	Survivors<br><49 11%<br>50-59 16%<br>60-69 40%<br>70+ 33%<br><br>Controls<br><49 30%<br>50-59 23%<br>60-69 23%<br>70+ 24%	NR
Sweden	Radiumhemmet, Karolinska University Hospital in Stockholm and	Treated 1991-2003	Baloch (2021)	247	Gynaecological	NR	16-29 1%,<br>30-49 27%,<br>50-64 72%	NR

Country of recruitment	Source of patients	Year of diagnosis / treatment	First Author (year of publication)	Sample size [1]	Cancer site	Extent of disease [2]	Age (years) [3]	Race / ethnicity [4]
	Jubileumskliniken, Sahlgrenska University Hospital in Gothenburg							
UK (England)	National Prostate Cancer Audit / English National Cancer Registry	Diagnosed 2014-2016	Nossiter (2020)	13,131	Prostate	Gleason Score<br>≤6 6.8%<br>7 57.3%<br>≥8 36%	<60 3.9%<br>61-70 32.8%<br>71-80 57%<br>>80 6.3%	White 95.9%
UK (England)	English National Cancer Registry	Diagnosed 2014-2016	Parry (2020)	5,468	Prostate	Non-metastatic, intermediate risk, high-risk or locally advanced	<60 4.7%<br>61-70 35.3%<br>71-80 54.1%<br>>80 5.9%	White 96.3%<br>Mixed 0.3%<br>Asian 1.2%<br>Black 1.5%<br>Other 0.8%
UK (England)	English National Cancer Registry	Treated 2010-2016	Parry (2021)	11,676	Prostate	Gleason Score<br>6 9.2%<br>7 (3 + 4) 31.1%<br>7 (4 + 3) 21.1%<br>8 17.1%<br>9 21.5%	<60 5.9%<br>60-70 35.6%<br>70-80 53.5%<br>>80 5%	NR
UK (England)	English National Cancer Registry	Diagnosed 2014-2016	Parry (2021)	13,259	Prostate	High risk 67.4%<br>Intermediate risk 32.6%	<60 4.2%<br>61-70 33.8%<br>71-80 56.1%<br>>80 6%	White 96%<br>Mixed 0.2%<br>Asian 1.3%<br>Black 1.8%<br>Other 0.6%
UK	Cancer registries of England, Scotland, Wales and Northern Ireland.	Diagnosed 1995-2015	Downing (2019)	35,823	Prostate	Stage at diagnosis<br>I/II 54.7%<br>III 20.1%<br>IV 14.2%	<55 1.8%<br>55-64 15.6%<br>65- 74 46.4%<br>75-84 30.9%<br>85+ 5.1%	NR

Country of recruitment	Source of patients	Year of diagnosis / treatment	First Author (year of publication)	Sample size [1]	Cancer site	Extent of disease [2]	Age (years) [3]	Race / ethnicity [4]
UK	Cancer registries of England, Scotland, Wales and Northern Ireland.	Treated 2008-2012	Mason (2022)	7,241	Prostate	Stage at diagnosis<br>I/II 56.9%<br>III 20.3%<br>IV 9%	<55 2%<br>55-64 17%<br>65- 74 49.5%<br>75-84 28.1%<br>85+ 3.4%	NR
USA	Prostate Cancer Outcomes Study (PCOS) - Surveillance, Epidemiology and End Results (SEER) registries	Diagnosed 1994-1995	Potosky (2001)	1,591	Prostate	Gleason score, RT<br>2-4 13%<br>5 21%<br>6 33%<br>7 24%<br>8-10 9%	At diagnosis<br>RT<br>55-59 6%<br>60-64 16%<br>65-69 31%<br>70-74 47%	RT<br>Hispanic<br>White 81%<br>Black 12%<br>Hispanic 7%
USA	Prostate Cancer Outcomes Study (PCOS) - Surveillance, Epidemiology and End Results (SEER) registries	Diagnosed 1994-1995	Hamilton (2001)	497 (EBRT)	Prostate	Gleason score<br>2-4 13.2%,<br>5 22.8%,<br>6 25%,<br>7 22.8%,<br>8-10 8.6%	At diagnosis<br><60 6.3%<br>60-64 10.7%<br>65-74 60.4%<br>75+ 22.7%	Non-Hispanic White 82%,<br>Black 10.2%,<br>Hispanic 8.1%
USA	Prostate Cancer Outcomes Study (PCOS) - Surveillance, Epidemiology and End Results (SEER) registries	Diagnosed 1994-1995	Hoffman (2003)	2,365 (total)<br>583 (RT)	Prostate	Gleason score<br>RT<br>2-4 34.8%<br>5-7 69%<br>8-10 9%	Mean (range)<br>66 (39-88)	Non-Hispanic White 74%<br>Non-Hispanic Black 13%<br>Hispanic 13%
USA	Prostate Cancer Outcomes Study (PCOS) - Surveillance, Epidemiology and	Diagnosed 1994-1995	Johnson (2004)	3,533 (total)<br>RT group 642 of which Non-Hispanic White 495	Prostate	Gleason score, RT, Non-Hispanic White<br>2-4 12.9%<br>5 21.1%<br>6 25.5%	At diagnosis<br>RT group, Non-Hispanic White <60 5.5%,<br>60-6 10.1%,<br>65-75 59.7%,	Non-Hispanic White 71%<br>African American 15%<br>Hispanic 13%

Country of recruitment	Source of patients	Year of diagnosis / treatment	First Author (year of publication)	Sample size [1]	Cancer site	Extent of disease [2]	Age (years) [3]	Race / ethnicity [4]
	End Results (SEER) registries			Completed baseline and 6 and/or 12-month follow-up		7 23.5%<br>8-10 17%	75+ 24.6%	
USA	Prostate Cancer Outcomes Study (PCOS) - Surveillance, Epidemiology and End Results (SEER) registries	Diagnosed 1994-1995	Potosky (2004)	1,187 (total at 5 years post-diagnosis)<br>286 (EBRT at 5 years post-diagnosis)	Prostate	Gleason score at 5-year survey,<br><br>EBRT<br>2-6 32%<br>7 40%<br>8-10 21%	At diagnosis<br><br>EBRT<br>55-59 31%<br>60-64 47%<br>65-69 31%<br>70-74 29%	At 5-year survey<br><br>EBRT<br>Hispanic 40%<br>Black 41%<br>White 31%
USA	Prostate Cancer Outcomes Study (PCOS) - Surveillance, Epidemiology and End Results (SEER) registries	Diagnosed 2011-2012	Resnick (2013)	1,655 (total)	Prostate	Gleason score RT<br>2-4 59.3%<br>5-7 22.1%<br>8-10 8.9%	Median 69(IQR 64-71) RT group	RT group<br><br>Non-Hispanic white 82%<br>No- Hispanic black 10.4%<br>Hispanic 7.7%
USA	Prostate Cancer Outcomes Study (PCOS) - Surveillance, Epidemiology and End Results (SEER) registries	Diagnosed 2011-2012	Hoffman (2017)	934 (total)<br>146 (RT)	Prostate	Gleason score RT<br>2-6 17.6%<br>7 22.3%<br>8-10 13.8%	At diagnosis, mean (SD), 62 (6.7)	Non-Hispanic White 80.2%<br>Non-Hispanic Black 9.7%,<br>Hispanic 10.1%
USA	CEASAR + CAPSURE (few) SEER registries	Diagnosed 2011-2012	Tyson (2016)	3,708 total, 503 [15]IMRT.<br>White 1,835,<br>African American 324,<br>Hispanic 179	Prostate	Gleason score<br><=6 52%<br>7 (3+4) 28%<br>7 (4+3) 10%<br>8+ 9%	Median (IQR) 64 (58-69)	White 22%<br>African American 32%<br>Hispanic 18%

Country of recruitment	Source of patients	Year of diagnosis / treatment	First Author (year of publication)	Sample size [1]	Cancer site	Extent of disease [2]	Age (years) [3]	Race / ethnicity [4]
USA	Surveillance, Epidemiology and End Results (SEER) registries	Diagnosed 2011-2012	Barocas (2017)	2,550 (total)<br>598 (RT)	Prostate	Gleason sore<br>6 (3 + 3) 52%<br>7 (3 + 4) 28%<br>7 (4 + 3) 10%<br>8-10 10%<br><br>Low risk 45%, Intermediate risk 39%, High risk 16%.	Mean (C.I.)<br>Total 63.8 (63.5,64.1)	White 77%
USA	Comparative Effectiveness Analysis of Surgery and Radiation Study (CAESAR) - Surveillance, Epidemiology and End Results (SEER) registries	Diagnosed 2011-2012	Lee (2018)	687	Prostate	Gleason score<br>≤6 34%<br>7 (3+ 4) 35%<br>7 (4 + 3) 14%<br>8, 9, 10 18%	Median (IQR)<br>69.0 (63 ,73)	NR
USA	CEASAR (including small number of CaPSURE) and PCOS), SEER registries	Diagnosed 2011-2012	O'Neill (2018)	943 (RT)	Prostate	Gleason score<br><br>IMRT<br>≤6 33.6%<br>7 (3 + 4) 33.6%<br>7 (4 + 3) 14.8%<br>8-10 18%<br><br>1990s EBRT<br>≤6 60.3%<br>7 (3 + 4) 7.8%<br>7 (4 + 3) 9.5%<br>8-10 6.3%	Contemporary<br><br>IMRT<br><55 4.9%<br>55-59 7.3%<br>60-64 15.6%<br>65-69 25.7%<br>70-74 27.2%<br>75+ 19.3%<br><br>1990s EBRT<br><55 4.6%<br>55-59 5.5%<br>60-64 11.6%<br>65-69 23.3%	Contemporary IMRT<br><br>Black 17.3%<br>Hispanic 4.9%<br>Other 4.1%<br>White 72.8%<br><br>1990s EBRT<br>Black 12.8%<br>Hispanic 10.1%<br>Other 0%<br>White 77.1%

Country of recruitment	Source of patients	Year of diagnosis / treatment	First Author (year of publication)	Sample size [1]	Cancer site	Extent of disease [2]	Age (years) [3]	Race / ethnicity [4]
							70-74 35.4%<br>75+ 20.6%	
USA	CEASAR + CAPSURE (few) SEER registries	Diagnosed 2011-2012	Tyson (2018)	2117	Prostate	NR	Median (IQR)<br><br>Low risk<br>62 (56-67)<br><br>Intermediate risk<br>64 (59-70)<br><br>High risk<br>65 (60-71)	Low risk<br>White 74%<br>African American 13%<br>Other 13%<br><br>Intermediate risk<br>White 76%<br>African American 14%<br>Other 10%<br><br>High risk<br>White 68%<br>African American 17%<br>Other 15%
USA	Comparative Effectiveness Analysis of Surgery and Radiation Study (CAESAR) - Surveillance, Epidemiology and End Results (SEER) registries	Diagnosed 2011-2012	Heulster (2020)	1312 ([16]RP only)<br>113 (RP+[17]sRT)	Prostate	Gleason score<br><br>RP only<br>6≤ 55%<br>7 (3 + 4) 29%<br>7 (4 + 3) 10%<br>8, 9, 10 6%<br><br>RP +sRT<br>6≤ 20%<br>7 (3 + 4) 38%<br>7 (4 + 3) 18%<br>8, 9, 10 24%	Median (IQR)<br><br>RP only<br>62 (57, 66)<br><br>RT+sRT<br>63 (58, 66)	RP only<br>White 77%<br>Black 12%<br>Hispanic 8%<br>Asian 3%<br>Other 1%<br><br>RT+sRT<br>White 75%<br>Black 10%<br>Hispanic 9%<br>Asian 4%<br>Other 2%
USA	Comparative Effectiveness	Diagnosed 2011-2012	Hoffman (2020)	2005 (Total)<br>261 (EBRT)	Prostate	Risk<br>Low	At diagnosis<br>Median 64	Non-Hispanic White 77%

Country of recruitment	Source of patients	Year of diagnosis / treatment	First Author (year of publication)	Sample size [1]	Cancer site	Extent of disease [2]	Age (years) [3]	Race / ethnicity [4]
	Analysis of Surgery and Radiation Study (CAESAR) - Surveillance, Epidemiology and End Results (SEER) registries			217 (EBRT + ADT)<br>363 ([18]AS)<br>402 (RP)		46%<br>Favourable intermediate<br>23%<br>Unfavourable intermediate<br>11%<br>High<br>20%	IQR 59-70	
USA	CEASAR (including small number of CaPSURE) and PCOS), SEER registries	Diagnosed 2011-2012	Pasalic (2021)	695	Prostate	Gleason score<br>≤6 34%<br>7 (3 + 4) 35%<br>7 (4 + 3) 14%<br>≥8 17%	At diagnosis, Median (IQR) 69 (63-73)	White 71%<br>Black 18%<br>Hispanic 6%<br>Asian 3%<br>Other 1%
USA	Comparative Effectiveness Analysis of Surgery and Radiation Study (CAESAR) - Surveillance, Epidemiology and End Results (SEER) registries	Diagnosed 2011-2012	De (2022)	1645<br>(112 EBRT-[19]LDR)	Prostate	Gleason score<br>EBRT-LDR<br></=6 34%, 3 + 4 36%, 4 + 3 11%, >8 20%<br><br>RP<br><6 49%, 3 + 4 30%, 4 + 3 11%, >/= 8 10%	At diagnosis, Mean (IQR), EBRT-LDR 66 (60, 71)<br><br>RP<br>62 (57, 66)	EBRT-LDR<br>White 74%, Black 21%, Hispanic 3%, Asian 1%, Other 2%,<br><br>RP<br>White 75%, Black 12%, Hispanic 8%, Asian 3%, Other 2%
USA	CEASAR + CAPSURE (few) SEER registries	Diagnosed 2011-2012	Wallis (2022)	587	Prostate	Gleason score<br><br>≤6 35%<br>7 (3 + 4) 34%<br>7 (4+ 3) 15%<br>8, 9, 10 16%	At diagnosis Mean (IQR) 69 (64-73)	White 70%<br>Black 18%<br>Hispanic 6%<br>Asian 4%<br>Other 1%

Country of recruitment	Source of patients	Year of diagnosis / treatment	First Author (year of publication)	Sample size [1]	Cancer site	Extent of disease [2]	Age (years) [3]	Race / ethnicity [4]
USA	North Carolina Central Cancer Registry	Diagnosed 2011-2013	Chen (2017)	1141(total)<br>249 (EBRT)<br>109 (BT)	Prostate	Gleason score %≤6, 7, 8-10<br><br>AS 86.2, 13.2, 0.6<br>EBRT 35.8, 49.2, 15<br>BT 79.219.8, 0.9<br>RP 45.6, 44.3, 10.1	At diagnosis<br>Mean (SD)<br>AS 67 (7.3)<br>EBRT 67 (7.1)<br>BT 66 (7.3)<br>RP 62 (6.8)	% White, African American, Other<br>AS 77, 22, 1<br>EBRT 62, 34, 4<br>BT 75, 24, 1<br>RP74, 24, 2
USA	North Carolina Central Cancer Registry	Diagnosed 2011-2013	Moon (2019)	189 EBRT<br>387 AS	Prostate	Localised	At diagnosis<br>Mean (SD)<br>EBRT 66 (6.9)<br>AS 66 (7.5)	% White<br>EBRT 66<br>AS 74
USA	Metropolitan Detroit Cancer Surveillance System	Diagnosed 1996-1998	Schwartz (2002)	398<br>115 (RT)	Prostate	Localised	At diagnosis<br>RT group<br>50-59 11.3%<br>60-69 40%<br>>70 48.7%	RT Group<br>Black 52.2%<br>White 45.2%<br>Other 2.6%

Alternating shading indicates groups of papers based on the same dataset. [1]Sample sizes are those at baseline for all respondents, unless otherwise stated. Response rates may differ across questions and over time for longitudinal studies. **[2]**Cancer details are for whole study population unless otherwise stated. **[3]**Race / ethnicity details are for whole study population unless otherwise stated. **[4]**Age, refers to age at baseline data collection (T1) for whole study population, unless otherwise stated. [5]External Beam Radiotherapy, [6]Confidence Interval, [7]Not Reported, [8]Androgen Deprivation Therapy, [9]Inter Quartile Range, [10]Standard Deviation, [11]Tumor, Nodes, Metastases, [12]Radiotherapy, [13]International Federation of Gynecology and Obstetrics, [14]Standard Error, [15]Intensity Modulated Radiotherapy, [16]Radical Prostatectomy, [17]salvage Radiotherapy, [18]Active Surveillance, [19]Low Dose-Rate

Supplementary Table S4. Outcome measures

Instrument	Citation	Dimensions assessed	Scoring
EPIC-26	Szymanski KM, Wei JT, Dunn RL, Sanda MG. Development and validation of an abbreviated version of the expanded prostate cancer index composite instrument for measuring health-related quality of life among prostate cancer survivors. Urology 2010;76(5):1245–50	Bowel function, bowel bother	0-100 scale, higher score represents better quality of life.
UCLA PCI	Litwin MS, Hays RD, Fink A, Ganz PA, Leake B, Brook RH. The UCLA Prostate Cancer Index: Development, re-liability, and validity of a health-related quality of life measure. Med Care 1998; 36(7): 1002–1012.	Bowel function, bowel bother	0-100 scale, higher score represents better quality of life
EORTC QLQ-C30	Aaronson et al 1993 QLQ-C30: A quality-of-life instrument for use in international clinical trials in oncology.	Scores for constipation, diarrhoea and overall bowel symptoms	0-100 scale, higher scores reflecting more symptoms
EORTC QLQ-PR25	George van Andel 2008 An international field study of the EORTC QLQ-PR25: A questionnaire for assessing the health-related quality of life of patients with prostate cancer	Bowel symptoms	0-100 scale, higher scores reflecting more symptoms
PCSI	Clark JA, Talcott JA. Symptom indexes to assess outcomes of treatment for early prostate cancer. Med Care. 2001;39(10):1118-1130.	Bowel problems, 6 domains (diarrhoea, urgency of bowel movements, pain during bowel movements, rectal bleeding, abdominal cramping, tenesmus)	Each domain is scored from 0 to 100, with a higher score indicating more or worse dysfunction
EPIC-50	Wei JT, Dunn RL, Litwin MS, Sandler HM, Sanda MG. Development and validation of the expanded prostate cancer index composite (EPIC) for comprehensive assessment of health-related quality of life in men with prostate cancer. Urology 2000; 56:899–905.	Bowel function, bowel bother	0-100 scale, higher score represents better quality of life.
EORTC QLQ-EN24	Greimel E, Nordin A, Lanceley A, et al. Psychometric validation of the European Organisation for Research and Treatment of Cancer Quality of Life Questionnaire-Endometrial Cancer Module (EORTC QLQ-EN24). Eur J Cancer2011; 47:183–90.	Gastrointestinal symptoms	0-100 scale, higher scores reflecting more symptoms

EPIC-26 = Expanded Prostate Cancer Index Composite; UCLA PCI = University of California Los Angeles Prostate Cancer Index; EORTC QLQ-C30 = European Organisation for Research and Treatment of Cancer Quality of Life Questionnaire-Core; EORTC QLQ-PR25 = European Organisation for Research and Treatment of Cancer Quality of Life Questionnaire-Prostate Cancer; PCSI = Prostate Cancer Symptom Indices; EPIC-50 = Expanded Prostate Cancer Index Composite; EORTC QLQ-EN24 = European Organisation for Research and Treatment of Caner Quality of Life Questionnaire-Endometrial Cancer

Supplementary Table S5. Quality appraisal

First Author (year)	Aim	Response Rate	Prospective collection of data	Measurement of bowel outcomes	Timing of data collection	Loss to follow-up	Prospective calculation of the study size	An adequate control group	Contemporary groups	Baseline equivalence of groups
	0: Not stated<br><br>1: Stated but does not include bowel symptoms<br><br>2: Stated aim includes assessment of colorectal symptoms	0: Not stated<br><br>1: Response rate of eligible patients stated<br><br>2: response rate and reasons for non-participation stated, or comparison between responders and non-responders	0: Cross sectional data at one point in time<br><br>1: Cross sectional data at multiple time points (different subjects)<br><br>2: longitudinal data for same subjects	0: no explanation of bowel outcome measure<br><br>1: survey tool created for the study or adapted from a standard/validated tool<br><br>2: standard / validated tool used as intended	0: no report of timing of data collection relative to treatment or diagnosis<br><br>1: timing of data collection reported relative to diagnosis but not relative to treatment<br><br>2: timing of data collection reported relative to treatment	0: no detail or cross sectional<br><br>1: longitudinal with partial detail on loss to follow-up (e.g. denominator for each time point)<br><br>2: Full detail of loss to follow-up stating how many died, how many could not be contacted and what the response rate was among those who were contacted.	0: no calculations<br><br>1: calculations provided but no detail or on different endpoints<br><br>2: calculations provided for 1 or more bowel symptom endpoints	0: No non-RT comparison group<br><br>1: Cancer comparison group<br><br>2: Non-cancer comparison group	0: historical controls<br><br>2: outcome in intervention and control assessed in same time period	0: not reported or unclear<br><br>1: Statistical support provided for assessment of baseline equivalence of potential confounders<br><br>2: Evidence that groups are similar with respect to age and at least 1 other potential confounder or multivariate analysis controls for age and at least 1 other potential confounder.
Baloch (2021)	1	2	0	1	2	0	0	NA	NA	NA
Bandarage (2016)	2	1	2	2	1	1	0	NA	NA	NA
Barocas (2017)	1	1	2	2	1	1	1[1]	1	2	2
Bergmark (2002)	1	1	0	1	1	0	0	1	2	0
Carlsson (2015)	2	2	0	1	1	0	0	2	2	2
Chen (2017)	1	1	2	2	2	1	0	1	2	2

First Author (year)	Aim	Response Rate	Prospective collection of data	Measurement of bowel outcomes	Timing of data collection	Loss to follow-up	Prospective calculation of the study size	An adequate control group	Contemporary groups	Baseline equivalence of groups
De (2022)	1	1	2	2	1	1	0	1	2	2
Downing (2019)	1	2	0	2	1	0	0	NA	NA	NA
Drummond (2015)	1	2	1	2	1	0	0	1	2	2
Dunberger (2010)	2	2	0	1	2	0	0	2	2	2
Dunberger (2009)	1	2	0	1	2	0	0	2	2	2
Fossa (2022)	2	1	0	0	2	0	0	NA	NA	NA
Gavin (2015)	1	2	0	2	1	0	0	1	2	2
Hamilton (2001)	2	2	2	1	1	1	1[2]	NA	NA	NA
Heulster (2020)	1	1	1	2	1	1	1[1]	1	2	2
Hoffman (2003)	1	1	0	0	1	1	1[2]	1	2	2
Hoffman (2017)	1	2	0	1	1	1	1[2]	1	2	2
Hoffman (2020)	1	1	1	2	1	1	1[1]	1	2	2
Johnson (2004)	2	1	2	1	1	1	1[2]	1	2	2
Kyrdalen (2012)	2	1	0	1	2	0	0	1	2	2
Lee (2018)	1	1	2	2	1	1	1[1]	NA	NA	NA
Lehto (2017)	1	1	0	1	1	0	0	1	2	2
Mason (2022)	1	1	2	2	1	1	0	1	2	2
Mazariego (2020)	2	1	2	2	1	1	0	2	2	2
Mols (2008)	2	2	0	2	1	0	0	1	2	2
Moon (2019)	1	0	0	2	1	0	0	1	2	2
Nguyen-Nielson (2019)	1	1	2	2	1	1	0	1	2	2
Nossiter (2020)	1	2	0	2	1	0	0	NA	NA	NA

First Author (year)	Aim	Response Rate	Prospective collection of data	Measurement of bowel outcomes	Timing of data collection	Loss to follow-up	Prospective calculation of the study size	An adequate control group	Contemporary groups	Baseline equivalence of groups
O'Neil (2018)	2	0	2	2	1	0	1[1]	NA	NA	NA
Ong (2022)	1	1	0	2	2	0	0	NA	NA	NA
Parry (2020)	1	1	0	2	1	0	0	NA	NA	NA
Parry (2020)	2	2	0	2	1	0	0	NA	NA	NA
Parry (2021)	1	1	0	2	1	0	0	NA	NA	NA
Pasalic (2021)	1	1	2	2	1	0	1[1]	NA	NA	NA
Potosky (2004)	1	2	2	1	1	1	1[2]	1	2	2
Potosky (2000)	2	1	2	1	1	2	1[2]	1	2	2
Pryor (2021)	1	1	0	2	2	0	0	NA	NA	NA
Resnick (2013)	2	1	2	1	1	1	0	1	2	2
Schwartz (2002)	2	2	2	1	2	1	0	2	2	2
Smith (2009)	1	2	2	2	1	1	0	2	2	2
Steineck (2017)	2	1	0	1	2	0	0	2	2	2
Tyson (2016)	2	0	2	2	1	0	1[1]	1	2	2
Tyson (2018)	1	0	2	2	1	0	1[1]	1	2	2
Van de Poll-Franse (2012)	1	2	0	2	0	0	0	1	2	2
Wallis (2021)	1	0	2	2	2	1	0	NA	NA	NA

[1] References the following methods paper: Barocas DA, Chen V, Cooperberg M, et al. Using a population-based observational cohort study to address difficult comparative effectiveness research questions: the CEASAR study.J Comp Eff Res. 2013;2(4):445-460

[2] References the following methods paper: Potosky AL, Harlan LC, Stanford JL, Gilliland FD, Hamilton AS, Albertsen PC, et al. Prostate cancer practice patterns and quality of life: the Prostate Cancer Outcomes Study. J Natl Cancer Inst 1999;91:1719–24.

Supplementary Table S6. Bowel outcome findings by symptom and paper

Bowel symptom	Indicator	Dataset	First Author (year)	Outcome measure(s)	Prevalence (radiotherapy patients)	Severity (radiotherapy patients)	Comparisons
**Impaired bowel function**	Bowel function score	CEASAR[1] & CAPSURE[2] (few) SEER[3] registries	Tyson (2016)	EPIC[4]-26			*Comparison of treatments (radiotherapy v other treatments)*<br><br>Adjusted mean difference in function between treatment types at 12 months (CI[5]) p-value<br><br>*White*<br>EBRT[6] vs AS[7] -2.9 $p<0.05$<br>RP[8] vs EBRT 4.2 (2.5, 5.9) $p<0.05$<br><br>*African American*<br>EBRT vs AS -3.6 (-7.8, 0.7)<br>RP vs EBRT -22.5 (-29.0, -16.0)<br><br>Difference in treatment effect, DID[9] (CI)<br>White vs African American<br>EBRT vs AS 0.6 (-3.5, 5.0)<br>RP vs EBRT (-4.0, 1.9)
	Bowel function score	CEASAR & CAPSURE (few) SEER registries<br><br>Diagnosed 2011-2012	O'Neil (2017)	EPIC-26 and UCLA PCI[10]			*Comparison of treatments (radiotherapy)*<br><br>At 6 months post-treatment, after adjusting for potential confounders, mean bowel function scores were more than 4 points higher (better) in patients who had received contemporary IMRT than in those who had received 1990s EBRT. At 12 months the difference was less than 1 point and the CI included zero.
	Bowel function score	CEASAR + CAPSURE (few) SEER registries<br><br>Diagnosed 2011-2012	Tyson (2018)	EPIC-26			*Comparison of treatments (radiotherapy v other treatments)*<br><br>Difference in difference between treatment and cancer severity<br><br>No clinically significant interactive effects between treatment (RP vs EBRT) and cancer severity (low-risk vs high-risk) for the bowel

Bowel symptom	Indicator	Dataset	First Author (year)	Outcome measure(s)	Prevalence (radiotherapy patients)	Severity (radiotherapy patients)	Comparisons
							irritative domain score (DID, 0.6 points [95% CI, −1.9 to 3.1]; p = 0.7)
	Bowel irritative domain score	Comparative Effectiveness Analysis of Surgery and Radiation Study (CAESAR) - Surveillance, Epidemiology and End Results (SEER) registries<br><br>Diagnosed 2011-2012	Heulster (2020)	EPIC-26		RP+sRT[11]<br>Median (IQR[12])<br>1 year 94 (88, 100)<br>3 years 94 (81, 100)<br>5 years 88 (75, 100)<br>(post-prostatectomy)	*Comparison of treatments (radiotherapy v other treatments)*<br><br>Comparison RP v RP+sRT (adjusted for covariates); score difference (CI) p-value<br>1 year 1.6 (-1.6, 4.9) p=0.323<br>3 years -3.3 (-7.7, 1.1) p=0.144<br>5 years -3.4 (-7.4, 0.5) p=0.088<br>(post-prostatectomy)
	Bowel function score	Comparative Effectiveness Analysis of Surgery and Radiation Study (CAESAR) - Surveillance, Epidemiology and End Results (SEER) registries<br><br>Diagnosed 2011-2012	Hoffman (2020)	EPIC-26		Bowel function score<br><br>Median (IQR)<br>Baseline 100 (96, 100)<br>6 months 96 (83, 100)<br>1 year 96 (83, 100)<br>3 years 100 (88, 100)<br>5 years 100 (88, 100)	*Comparison of treatments (radiotherapy v other treatments)*<br><br>EBRT v AS:<br>OR (CI) p-value<br>6 months -3.7 (-5.8, - 1.7) p<.001<br>1 year -3.5 (-5.1, -1.8) p<.001<br>3 years -2.9 (-4.9, -0.9) p=.005<br>5 years -2.7 (-5.0, -0.5) p=.02<br><br>No clinically meaningful difference between EBRT and AS at any point in time.
	Bowel function score	Surveillance, Epidemiology and End Results (SEER) registries<br><br>Diagnosed 2011-2012	De (2022)	EPIC-26		Median baseline 100<br>Median 5 years 92<br>(clinically significant)	*Comparison of treatments (radiotherapy v other treatments)*<br><br>No decline in score in RP group<br><br>EBRT-LDR[13] v RP<br>Point difference in score<br>6 months -7.1 (p<0.001)<br>1 Year -9.1 (p<0.001)<br>3 years -6.3 (p<0.001)<br>5 years -4.0 (p=0.006)
	Bowel function score	CEASAR + CAPSURE (few) SEER registries<br><br>Diagnosed 2011-2012	Wallis (2022)	EPIC-26		Median (IQR)<br><br>*Prostate and pelvic RT*<br>Baseline 100 (88, 100)	*Comparison of treatments (radiotherapy)*<br><br>Multivariable adjusted model, prostate and pelvic RT vs prostate only RT

Bowel symptom	Indicator	Dataset	First Author (year)	Outcome measure(s)	Prevalence (radiotherapy patients)	Severity (radiotherapy patients)	Comparisons
						6 months 100 (79, 100)<br>1 year 96 (83, 100)<br>3 years 96 (84, 100)<br>5 years 96 (83, 100)<br><br>*Prostate only RT*<br>Baseline 100 (92, 100)<br>6 months 96 (83, 100)<br>1 year 96 (83, 100)<br>3 years 96 (83, 100)<br>5 years 96 (88, 100)	Effect estimate (CI)<br><br>6 months 0.94 (-2.60, 4.49) p=0.6<br>1 year 2.02 (-0.75, 4.79) p=0.15<br>3 years 3.07 (-0.38, 6.52) p=0.081<br>5 years 0.76 (-3.38, 4.90) p=0.72
	Bowel function score	National Prostate Cancer Audit / English National Cancer Registry<br><br>Diagnosed 2014-2016	Nossiter (2020)	EPIC-26			*Comparison of treatments (radiotherapy)*<br>No significant difference in bowel function between the RT[14] groups.<br>Bowel Mean (SD[15])<br>CRT[16] vs HRT[17]<br>85.7 (18.4); 86.2 (18.3)
	Bowel function score	English National Cancer Registry<br><br>Diagnosed 2014-2016	Parry (2020)	EPIC-26		Mean (SD)<br>PO-IMRT[18] 85.6 (18.4)<br>PPLN-IMRT[19] 85.5 (18.6)	*Comparison of treatments (radiotherapy)*<br><br>Pelvis Only IMRT v Pelvis and Lymph node IMRT, no significant difference in mean bowel function scores. Adjusted difference in means (CI) 0.2 (-1.1, 1.5)
	Bowel function score	English National Cancer Registry<br><br>Diagnosed 2014-2016	Parry (2021)	EPIC-26		Mean (CI)<br>EBRT monotherapy 84.4 (84.1, 84.8)<br>HDR-BB 85.8 (84.4, 87.2)	*Comparison of treatments (*radiotherapy*)*<br>No sig diff v BB[20]
	Bowel function score	English National Cancer Registry<br><br>Treated 2010-2016	Parry (2021)	EPIC-26		Mean (SD)<br>EBRT monotherapy 85.9 (18.3)<br>HDR[21]-BB 87 (17.2)	*Comparison of treatments (radiotherapy)*<br><br>No significant difference in bowel function score when comparing patients treated with HDR-BB and those treated with EBRT monotherapy. Adjusted difference in means (CI) p-value<br>1.4 (-0.7-3.5) p=0.206
	Bowel function score	Cancer registries of England, Scotland, Wales and Northern Ireland.	Downing (2019)	EPIC-26		(Adj mean, 95% CI)<br>Brachytherapy 88.8 (87.9-89.7)	*Comparison of treatments (radiotherapy v other treatments)*

Bowel symptom	Indicator	Dataset	First Author (year)	Outcome measure(s)	Prevalence (radiotherapy patients)	Severity (radiotherapy patients)	Comparisons
		Diagnosed 1995-2015				EBRT 86.2 (85.4-87.0)<br>EBRT and ADT[22] 84.4 (84.0 - 84.9)<br>Systemic therapy and EBRT 83.8 (82.1-85.5)	Mean bowel function score, 95% CI<br>Active surveillance 93·1 (92·6–93·6)<br>Watchful waiting 93·1 (92·6–93·7)<br>Surgery 93·4 (93·1–93·7)<br>ADT 90·9 (90·3–91·5)<br>Systemic therapy and ADT 90·7 (89·6–91·9)
	Bowel Function score	Cancer registries of England, Scotland, Wales and Northern Ireland.<br><br>Diagnosed 1995-2015	Mason (2022)	EPIC-26		Mean score, T1, T2<br>Initial treatment<br>Surgery and EBRT (+-ADT) T1 86.0; T2 87.2<br>EBRT T1 86.6 T2 87.1<br>EBRT and ADT T1 84.5 T2 84.9<br>Systemic therapy and EBRT(+-ADT) T1 85.3 T2 84.1<br><br>Additional Treatment<br>EBRT/BT T1 92.9 T2 84.6<br>Surgery to surgery and EBRT(+-ADT) T1 93.4 T2 86.7<br>EBRT to EBRT and ADT T1 86.4 T2 87.4<br>EBRT and ADT to surgery and EBRT (+-ADT) 82.2 T2 82.0<br>EBRT and ADT to systemic therapy and EBRT T1 82.1 T2 82.2<br>ADT to EBRT and ADT	*Comparison of treatments (radiotherapy v other treatments)*<br><br>Worse bowel function reported by men whose first active treatment included EBRT (alone or combined with ADT) or Brachytherapy compared with men whose first active treatment did not include radiotherapy. Clinically Meaningful Differences in mean bowel function scores were observed at follow-up (−8.3 points).<br><br>Men who reported the addition of EBRT reported worse bowel function at follow-up compared with men whose additional treatment did not include radiotherapy, with a Clinically Meaningful Differences for men treated with EBRT following initial ADT (−5.8 points) and men treated with combined EBRT and ADT following surgery (−6.7 points)
	Bowel bother score	New South Wales Central Cancer Registry<br><br>Diagnosed 2000-2002	Mazariego (2020)	Subset of items common to both UCLA-PCI and EPIC-26			*Comparison of treatments (radiotherapy v other treatments)*<br><br>EBRT had worse bowel bother score than controls at 6 months, and at 1, 2, 3, 5, and 10

Bowel symptom	Indicator	Dataset	First Author (year)	Outcome measure(s)	Prevalence (radiotherapy patients)	Severity (radiotherapy patients)	Comparisons
							years. The difference was clinically significant at all time points.
	Bowel function score	New South Wales Central Cancer Registry<br><br>Diagnosed 2000-2002	Smith (2009)	UCLA PCI		Mean bowel function score (SD)<br><br>EBRT<br>T1 49.3 (9.9)<br>T2 46.5 (10)<br><br>EBRT/ADT<br>T1 46.7 (11.3)<br>T2 44.1 (11.7)	
	Bowel problems score	North Carolina Central Cancer Registry<br><br>Diagnosed 2011-2013	Chen (2017)	Prostate Cancer Symptom indices (PCSI)		Bowel problems mean scores (CI), baseline, 3 months, 12 months, 24 months:<br>EBRT 5.7(4.6-6.7), 12 (9.8-14.3), 9 (7.3-10.7), 9.9 (7.4-12.4);<br>BT 6.1 (4.5-7.6), 9 (6.9-11.1), 7 (5.2-8.8), 6.8 (4.9-8.6)	*Comparison of treatments (radiotherapy v other treatments)*<br><br>Difference from AS, mean difference (CI): baseline, 3 months, 12 months, 24 months. EBRT worse at 3 months but no different by 24 months. Brachytherapy no difference.<br><br>EBRT -0.2 (-1.6, 1.1), 4.9 (2.4-7.4), 1.8 (-0.3-3.9), 3.7 (1.0-6.4)<br>BT 0.2 (-1.6-2), 1.9 (-0.5 - 4.3), -0.2 (-2.4 - 2), 0.6 (-1.5 - 2.7).
	Bowel problems score	North Carolina Central Cancer Registry<br><br>Diagnosed 2011-2013	Moon (2019)	Prostate Cancer Symptom Indices (PCSI)		Mean (SD)<br><br>Baseline 5.8 (12.1)<br>3 months 11.9 (21.2)<br>12 months 9.5 (19)<br>24 months 9.7 (21.4)	*Comparison of treatments (radiotherapy v other treatments)*<br><br>Mean difference score vs AS<br><br>Mean (CI)<br>Baseline -0.3 (-1.8-1.1)<br>3 months 4.6 (2, 7.3) (statistically significant)<br>12 months 2 (-0.5, 4.5)<br>24 months 3.2 (0.2, 6.2) (statistically significant)<br><br>EBRT significantly worse bowel problem score compared with AS at 3 months and 24 months. But stereotactic body radiotherapy no different.

Bowel symptom	Indicator	Dataset	First Author (year)	Outcome measure(s)	Prevalence (radiotherapy patients)	Severity (radiotherapy patients)	Comparisons
	Bowel symptoms	National Cancer Registry Ireland & Northern Ireland Cancer Registry<br><br>Diagnosed 1995-2010	Drummond (2015)	EORTC[23] QLQ[24]-30 and EORTC QLQ-PR25[25]		Unadjusted mean score EORTC:<br>EBRT+ADT 9.3<br>EBRT 9.1<br>BT[26] 4.3	*Comparison of treatments (radiotherapy v other treatments)*<br><br>Multivariate analysis comparing with RP: Difference (CI):<br>EBRT+ADT 3.23 (1.76, 4.71)<br>EBRT 3.75 (2.53, 4.98)<br>BT 0.12 (-2.24, 2.49)<br>EBRT and EBRT+ADT had significantly worse bowel symptom scores than RP - but not clinically significant.
	Bowel domain summary score	Cancer Registry of Norway<br><br>Diagnosed 2004-2015	Fossa (2022)	EPIC-26		Bowel Domain Summary Score mean (SD) 83.3 (19.1),	Mean (SD) Non-cancer group 93.0 (12.5), Statistically and clinically significant difference between cancer and non-cancer group after adjusting for age.
	Bowel summary score (max=100) and change from baseline	Prostate Cancer Outcomes Study (PCOS) - Surveillance, Epidemiology and End Results (SEER) registries	Hamilton (2001)<br><br>Diagnosed 1994-1995	Study specific		Bowel summary score:<br>Before diagnosis 92<br>6 months -12.6<br>12 months -6.4<br>24 months -5<br><br>Baseline v 24 months p≤.0001<br><br>At 24 months patients treated with radiotherapy showed a statistically significant mean decline in bowel function score of 5 points compared with baseline.	
	Bowel functioning score	Eindhoven Cancer Registry<br><br>Diagnosed 1994-1998	Mols (2008)	EPIC-26		Mean (SD)<br>87.8 (13.1)	*Comparison of treatments (radiotherapy v other treatments)*<br><br>Mean (SD)

Bowel symptom	Indicator	Dataset	First Author (year)	Outcome measure(s)	Prevalence (radiotherapy patients)	Severity (radiotherapy patients)	Comparisons
							RP 93.5 (9.4)<br>HT[27] 92.7 (9.5)<br>WW[28] 92.8 (10)<br><br>RT worse bowel functioning than other treatment groups, p<0.001, clinically significant.
	Bowel Summary Score					Mean (SD)<br>85.8 (14.3)	Mean (SD)<br>RP 92.8 (10.4)<br>HT 91.8 (10.2)<br>WW 92.5 (10.1)<br><br>RT worse bowel summary score than other treatment groups and from normative population, p<0.001, clinically significant.
	Bowel bother score					Mean (SD<br>84 (17.3	*Comparison of treatments (radiotherapy v other treatments)*<br><br>Mean (SD)<br><br>RP 92.5 (11.3)<br>HT 90.7 (12.9)<br>WW 92.6 (11.4)<br><br>RT worse bowel summary score than other treatment groups, p<0.001, clinically significant.
	Bowel function score	Danish Prostate Cancer Registry<br><br>Diagnosed 2010-2016	Nguyen-Neilsen (2019)	EPIC-26		Mean (read from graph)<br>400 days post-diagnosis<br><br>RT+ADT 65<br><br>800 days post-diagnosis	*Comparison of treatments (radiotherapy v other treatments)*<br><br>Mean (read from graph)<br><br>400 days post-diagnosis

Bowel symptom	Indicator	Dataset	First Author (year)	Outcome measure(s)	Prevalence (radiotherapy patients)	Severity (radiotherapy patients)	Comparisons
						RT+ADT 66	AS 74<br>RP 73<br>WW 71<br>Palliation 69<br><br>800 days post-diagnosis<br>AS 74<br>RP 72<br>WW 72<br>Palliation 69<br><br>Bowel function was most negatively affected by radiation, with or without adjuvant endocrine therapy (compared with Active Surveillance, Watchful Waiting, Palliation or Radical Prostatectomy) . Interaction effect of time and treatment type, estimate (CI) for RT group −5.3 (−6.71, −3.92) using baseline and AS group as reference, p<0.001 for trend over time and heterogeneity between treatment groups
	Bowel function score	Victorian Prostate Cancer Registry<br><br>Diagnosed 2015-2020	Ong (2022)	EPIC-26		median (IQR) 95.8 (83.3-100), Mean (SD) 89.3 (15.7)	*Comparison of treatments (radiotherapy)*<br><br>No significant difference in score between EBRT and EBRT + BT (BT sample <100)
	Bowel function score	Australia and New Zealand Prostate Cancer Outcomes Registry PCOR-ANZ<br><br>Treated 2016-2019	Pryor (2022)	EPIC-26		CRT group: Median (IQR) 92 (79 to 100), Mean (SD) 86.2 (18.3), HRT group: Median (IQR) 96 (83 to 100), Mean (SD) 88.1 (16.1)	
	Gastrointestinal symptom score	Eindhoven Cancer Registry<br><br>Diagnosed 1999-2007	Van de Poll-Franse (2012)	EORTC QLQ-EN[29]25 plusQLQ-30 item on diarrhoea		Mean (SD)<br>RT only 23 (21)	*Comparison of treatments (radiotherapy v other treatments)*<br><br>Mean (SD)<br>No Lymphoademectomy (LA[30]) No RT (N=377) 16 (17) vs LA no RT (N=198) 17

Bowel symptom	Indicator	Dataset	First Author (year)	Outcome measure(s)	Prevalence (radiotherapy patients)	Severity (radiotherapy patients)	Comparisons
							(18) vs RT only (N=153) 23 (21) vs LA plus RT (N=14) 23 (20) adjusted p-value 0.17<br><br>RT vs No LA no RT p=0.04 adjusted for possible confounders<br><br>Mean (SD)<br>LA, no RT 17 (18)<br>No LA no RT 16 (17)<br><br>Patients treated with pelvic radiotherapy had a somewhat higher mean gastrointestinal symptom score than those whose treatment did not include pelvic radiotherapy.
**Bowel function bother**	Bowel function bother	Surveillance, Epidemiology and End Results (SEER) registries<br><br>Diagnosed 2011-2012	Barocas (2017)	EPIC-26			*Comparison of treatments (radiotherapy v other treatments)*<br><br>EBRT vs AS - EBRT had greater odds of a moderate or big bother with bowel function, OR 2.8, (CI 1.6, 5.2), p<0.0001 at 6 months – adjusted for baseline (not clinically significant) No sig diff at 12 or 36 months or at any time point compared with RP.
	Bowel Function Problem	Comparative Effectiveness Analysis of Surgery and Radiation Study (CAESAR) - Surveillance, Epidemiology and End Results (SEER) registries<br><br>Diagnosed 2011-2012	Lee (2018)	EPIC-26	*EBRT+BT*<br>Baseline 3%<br>6 months 6%<br>1 year 4%<br>3 years 8%<br><br>*EBRT*<br>Baseline 4%<br>6 months 8%<br>1 year 8%<br>3 years 6%		

Bowel symptom	Indicator	Dataset	First Author (year)	Outcome measure(s)	Prevalence (radiotherapy patients)	Severity (radiotherapy patients)	Comparisons
	Bowel function bother	Comparative Effectiveness Analysis of Surgery and Radiation Study (CAESAR) - Surveillance, Epidemiology and End Results (SEER) registries<br><br>Diagnosed 2011-2012)	Hoffman (2020)	EPIC-26	% Moderate/big problem<br>EBRT<br>Baseline 2%<br>6 months 4%<br>1 year 7%<br>3 years 4%<br>5 years 4%		*Comparison of treatments (radiotherapy v other treatments)*<br><br>Comparison EBRT v AS: Odds of moderate or big problem<br>OR[31] (CI) p-value<br><br>6 months 2.4 (1, 5.8) p=.05<br>1 year 1.8 (0.9, 3.4) p=.08<br>3 years 1 (0.4, 2.8) p=.98<br>5 years 1.2 (0.5, 3.2) p=.68<br><br>No clinically meaningful difference between EBRT and AS at any point in time.
	Bowel function bother	CEASAR (including small number of CaPSURE) and Prostate Cancer Outcomes Study (PCOS), SEER registries<br><br>Diagnosed 2011-2012	Pasalic (2021)	EPIC-26	Moderate or big problem<br><br>EBRT-LDR<br>Baseline 3%<br>6 months 6%<br>1 year 4%<br>3 years 7%<br>5 years 4%<br><br>EBRT<br>Baseline 4%<br>6 months 8%<br>1 year 8%<br>3 years 6%<br>5 years 5%		*Comparison of treatments (radiotherapy)*<br><br>EBRT-LDR v EBRT<br>Effect, (CI), p-value<br><br>6 months 0.5 (0.2-1.5) p=0.24<br>1 year 0.8 (0.4-1.8) p=0.59<br>3 years 1.6 (0.6-3.7) p=0.42<br>5 years 0.9 (0.2-3.3) p=0.83
	Bowel function bother	Surveillance, Epidemiology and End Results (SEER) registries<br><br>Diagnosed 2011-2012	De (2022)	EPIC-26	% Moderate/big problem<br>EBRT-LDR<br>Baseline 3%<br>6 months 6%<br>1 year 4%<br>3 years 7%<br>5 years 4%	-	*Comparison of treatments (radiotherapy v other treatments)*<br><br>EBRT-LDR v RP<br><br>OR moderate or big problem (p-value)<br>0.9 (0.853)<br>1.4 (0.393)<br>2.4 (,0.086)

Bowel symptom	Indicator	Dataset	First Author (year)	Outcome measure(s)	Prevalence (radiotherapy patients)	Severity (radiotherapy patients)	Comparisons
							1.0 (0.947)
	Bowel problems	Cancer Registry of Norway<br><br>Diagnosed 2004-2015	Fossa (2022)	EPIC-26	% Moderate/big problem<br><br>12% (read from graph)		*Comparison against non-cancer group*<br><br>% Moderate/big problem<br><br>4% (read from graph) No statistical support presented
	Intestinal irritative symptoms	Cancer Registry of Norway and Norwegian Prescription Database<br><br>Diagnosed 2004	Kyrdalen (2012)	Selected items from EPIC-50	RT 47%<br>RT+HT 42%		*Comparison of treatments (radiotherapy v other treatments)*<br><br>No treatment 21%<br><br>RT or RT+HT v No treatment or RP, p<.01, adjusted for age, risk and comorbidities.<br><br>The proportion of prostate cancer patients with intestinal irritative symptoms was greater in those treated with radiotherapy than those receiving surgery or no-treatment.
	Bowel syndromes	Radiumhemmet, Karolinska University Hospital in Stockholm and Jubileumskliniken, Sahlgrenska University Hospital in Gothenburg<br><br>Treated 1991-2003	Baloch (2021)	Study specific	Number of syndromes experienced<br><br>0 53%<br>1 20%<br>2 20%<br>3 7%		
	Bowel dysfunction bother	Victorian Prostate Cancer Registry<br><br>Diagnosed 2009-2014	Bandarage (2016)	EPIC-26	T1 4% (Big bother)<br>T2 5.1% (Big bother)		
	Bowel dysfunction	Swedish National Prostate Cancer Registry<br><br>Diagnosed 1997-2002	Carlsson (2018)	Adapted from Prostate Cancer Symptom Scale			*Comparison of treatments (radiotherapy v no cancer)*<br><br>Bowel Dysfunction (any) - OR and 95% CI for comparisons of RT against no prostate cancer control group. RT 2.46 (1.73-3.49)

Bowel symptom	Indicator	Dataset	First Author (year)	Outcome measure(s)	Prevalence (radiotherapy patients)	Severity (radiotherapy patients)	Comparisons
							RP and AS groups showed no difference from no PC
	Bowel problems (current)	National Cancer Registry Ireland & Northern Ireland Cancer Registry<br><br>Diagnosed 1995-2010	Gavin (2015)	EORTC QLQ-30 and PR-25, EQ5D-5L, DASS 21 and Decisional Regret scale	% (CI)<br>EBRT+HT 22 (18.7,25.6),<br>EBRT 19.2 (16.9,21.6), BT 7.3 (3.4,13.4),		*Comparison of treatments (radiotherapy v other treatments)*<br><br>% (CI)<br>RP 9.2 (7.4,11.3),<br>WW/AS 2.4 (0.7,6.1).<br><br>EBRT+HT highest proportion current bowel problems
	Bowel function problem	Prostate Cancer Outcomes Study (PCOS) - Surveillance, Epidemiology and End Results (SEER) registries<br><br>Diagnosed 1994-1995	Hamilton (2001)	Study specific	*Moderate - big*<br>Before diagnosis 6.7%<br>6 months 13.5%<br>12 months 9.7%<br>24 months 8.9%<br><br>*Small*<br>Before diagnosis 23.3%<br>6 months 40.8%<br>12 months 32.3%<br>24 months 31.6%<br><br>*None*<br>Before diagnosis 69.9%<br>6 months 45.7%<br>12 months 56.8%<br>24 months 58.5%<br><br>Baseline v 24 months p≤.005		
	Bowel bother	Prostate Cancer Outcomes Study (PCOS) - Surveillance, Epidemiology and End Results (SEER) registries	Hoffman (2017)	NR	None 46.7%,<br>Very small/small 37.6%,<br>Moderate/big 15.6%		*Comparison of treatments (radiotherapy v other treatments)*<br><br>Bowel bother

Bowel symptom	Indicator	Dataset	First Author (year)	Outcome measure(s)	Prevalence (radiotherapy patients)	Severity (radiotherapy patients)	Comparisons
		Diagnosed 2011-2012					*Surgery*<br>None 65.7%<br>Very small/small 29.3%<br>Moderate/big 5%<br><br>*Conservative*<br>None 58.2%<br>Very small/small 28.9%<br>Moderate/big 13%<br><br>Surgery group least likely to report big bowel bother.
	Bowel movement problem	Prostate Cancer Outcomes Study (PCOS) - Surveillance, Epidemiology and End Results (SEER) registries<br><br>Diagnosed 1994-1995	Johnson (2004)	Adapted UCLA Prostate Cancer Index	Non-Hispanic White<br><br>*Baseline*<br>No 67%<br>Small 25.3%<br>Moderate/big 7.1%<br><br>*6 months*<br>No 43.5%<br>Small 39.9%<br>Moderate/big 15.7%<br><br>*12 months*<br>No 54.6%<br>Small 34%<br>Moderate/big 10%<br><br>*24 months*<br>No 55.3%<br>Small 34.7%<br>Moderate/big 8.6%<br><br>*60 months*<br>No 53.2%<br>Small 39.7%<br>Moderate/big 6.5%		

Bowel symptom	Indicator	Dataset	First Author (year)	Outcome measure(s)	Prevalence (radiotherapy patients)	Severity (radiotherapy patients)	Comparisons
	Bothered by frequency, pain or urgency	Prostate Cancer Outcomes Study (PCOS) - Surveillance, Epidemiology and End Results (SEER) registries<br><br>Diagnosed 1994-1995	Potosky (2000)	Adapted	Big /Moderate<br>8.4%		*Comparison of treatments (radiotherapy v other treatments)*<br><br>OR (CI) RP v RT 0.68 (0.31, 1.5)<br><br>The odds of bother from frequency, pain or urgency was higher in patients treated with radiotherapy than those treated with surgery, but the confidence interval included 1.
	Bowel Function Bother	Australia and New Zealand Prostate Cancer Outcomes Registry PCOR-ANZ<br><br>Treated<br>2016-2019	Pryor (2022)	EPIC-26	CRT group:<br>no/small problem<br>88.8% moderate/big problem 11.2%.<br>HRT group:<br>no/small problem<br>90.2% moderate/big problem 9.8%		
	Bothered by frequency, pain or urgency	Prostate Cancer Outcomes Study (PCOS) - Surveillance, Epidemiology and End Results (SEER) registries<br><br>Diagnosed 2011-2012	Resnick (2013)	Adapted	Big / moderate problem<br><br>2 years 7.9%<br>5 years 5.8%<br>15 years 16%		*Comparison of treatments (radiotherapy v other treatments)*<br><br>OR (CI) RP v RT<br><br>2 years 0.37 (0.14, 0.96)<br>5 years 0.93 (0.27, 3.22)<br>15 years 0.29 (0.11, 0.78)<br><br>The odds of bother from frequency, pain or urgency was greater in patients treated with radiotherapy than those treated with surgery at 2, 5 and 15 years, but at the 5-year timepoint the confidence interval included 1.
	Positive bother	Metropolitan Detroit Cancer Surveillance System<br><br>Diagnosed 1996-1998	Schwartz (2002)	Study specific / adapted	T1 (baseline) 0.8%<br>T2 2.3%<br>P=0.317		*Comparison of treatments (radiotherapy v other treatments)*<br><br>Comparison at follow-up of those reporting no bother at baseline<br>RP 1.6%<br>RT 4.6%<br>P=0.194

Bowel symptom	Indicator	Dataset	First Author (year)	Outcome measure(s)	Prevalence (radiotherapy patients)	Severity (radiotherapy patients)	Comparisons
	Bowel problems	New South Wales Central Cancer Registry<br><br>Diagnosed 2000-2002	Smith (2009)	UCLA PCSI	Moderate or big problems<br><br>EBRT<br>T1 30.2%<br>T2 14.5%<br><br>EBRT/ADT<br>T1 6%<br>T2 12.5%		*Comparison of treatments (radiotherapy v other treatments)*<br><br>Men who had EBRT had worse bowel function than controls at one year (OR 0.51, 95% CI 0.34 to 0.74) and at three years (OR 0.58, 95% CI 0.39 to 0.86) after diagnosis. Bowel bother affected in all treatment groups but worse affected in EBRT alone at one year (OR 0.24, 95% CI 0.15 to 0.36) and three years (OR 0.22, 95% CI 0.14 to 0.34) or in combination with ADT at one year (OR 0.24, 95% CI 0.16 to 0.35) and three years (OR 0.19 95% CI 0.13 to 0.28).
**Urgency**	Urgency bother	Surveillance, Epidemiology and End Results (SEER) registries<br><br>Diagnosed 2011-2012	Barocas (2017)	EPIC-26			*Comparison of treatments (radiotherapy v other treatments)*<br><br>EBRT vs RP - EBRT had greater odds of a moderate or big bother with urgency, OR 0.3, (CI 0.2,0.6), p<0.0001 at 3 years – adjusted for baseline (not clinically significant) No difference when EBRT vs AS
	Problem with urgency	Comparative Effectiveness Analysis of Surgery and Radiation Study (CAESAR) - Surveillance, Epidemiology and End Results (SEER) registries<br><br>Diagnosed 2011-2012	Lee (2018)	EPIC-26	*EBRT+BT*<br>Baseline 3%<br>6 months 10%<br>1 year 8%<br>3 years 10%<br><br>*EBRT*<br>Baseline 4%<br>6 months 8%<br>1 year 7%<br>3 years 7%		
	Urgency bother	Comparative Effectiveness Analysis of Surgery and Radiation Study (CAESAR) - Surveillance,	Hoffman (2020)	EPIC-26	% Moderate/big problem<br>EBRT		*Comparison of treatments (radiotherapy v other treatments)*

Bowel symptom	Indicator	Dataset	First Author (year)	Outcome measure(s)	Prevalence (radiotherapy patients)	Severity (radiotherapy patients)	Comparisons
		Epidemiology and End Results (SEER) registries<br><br>Diagnosed 2011-2012			Baseline 2%<br>6 months 4%<br>1 year 7%<br>3 years 7%<br>5 years 8%		Comparison EBRT v AS: Odds of moderate or big problem<br>OR (CI) p-value<br><br>6 months 1.7 (0.7, 3.9) p=0.23<br>1 year 1.8 0.9, 3.5) p=0.9<br>3 years 1.8 (0.8, 4.3) p=0.18<br>5 years1.4 (0.6, 3.2) p=0.42<br><br>No clinically meaningful difference between EBRT and AS at any point in time.
	Bowel urgency	CEASAR (including small number of CaPSURE) and Prostate Cancer Outcomes Study (PCOS), SEER registries<br><br>Diagnosed 2011-2012	Pasalic (2021)	EPIC-26	Moderate or big problem<br><br>EBRT-LDR<br>Baseline 3%<br>6 months 10%<br>1 year 8%<br>3 years 10%<br>5 years 6%<br><br>EBRT<br>Baseline 4%<br>6 months 8%<br>1 year 7%<br>3 years 7%<br>5 years 8%		*Comparison of treatments (radiotherapy)*<br><br>EBRT-LDR v EBRT<br>Effect, (CI), p-value<br><br>6 months 1.4 (0.5-3.8) p=0.46<br>1 year 1.9 (0.8-4.3) p=0.14<br>3 years 2.3 (0.9-6.1) p=0.094<br>5 years 1.2 (0.3-3.9) p=0.81<br><br>There was no statistically significant difference in the prevalence of bowel urgency between radiotherapy treatment groups at any time point from 6 months to 5 years post treatment.
	Urgency bother	Surveillance, Epidemiology and End Results (SEER) registries<br><br>Diagnosed 2011-2012	De (2022)	EPIC-26	Moderate or big problem<br><br>Baseline 3%<br>6 months 10%<br>1 year 8%<br>3 years 10%<br>5 years 6%		*Comparison of treatments (radiotherapy v other treatments)*<br><br>EBRT-LDR v RP<br><br>OR Moderate or big problem (p-value)<br><br>6 months 2.6 (0.038)<br>1 year 3.4 (0.002)<br>3 years 4.4 (<0.001)<br>5 years 2.4 (0.116)

Bowel symptom	Indicator	Dataset	First Author (year)	Outcome measure(s)	Prevalence (radiotherapy patients)	Severity (radiotherapy patients)	Comparisons
	Urgency distress	All (7) Swedish departments of gynaecological oncology<br><br>Diagnosed 1991-1992	Bergmark 2002	Study specific	(distress)<br>% much, moderate/much, a little/moderate/much in patients having internal or external RT +/- surgery<br><br>RT – much 17%, moderate or much 31%, any 44%		*Comparison of treatments (radiotherapy v other treatments)*<br><br>Surgery – much 3%, moderate or much 10%, any 19%<br><br>Urgency distress was more commonly reported by patients who had been treated with pelvic radiotherapy than those treated with surgery alone (statistical support for this association was not reported).
	Urgency at least 1 x per week in past 6 months	Karolinska University Hospital, Stockholm and Jubileumskliniken, Sahlgren University Hospital, Gothenburg<br><br>Treated 1991-2003	Dunberger (2009)	Study specific	29%		*Comparison against non-cancer group*<br><br>6%<br>RR 5.9 (3.3, 9.4) comparing with no cancer
	Urgency	Radiumhemmet, Karolinska University Hospital in Stockholm and Jubileumskliniken, Sahlgrenska University Hospital in Gothenburg<br><br>Treated 1991-2003	Steineck (2017)	Study specific	30%		*Comparison against non-cancer group*<br><br>One of 5 syndromes identified by the authors for which the distribution of scores differed between patients and non-cancer comparators, with scores being generally higher among patients. The cut-off for defining the syndrome was such that syndromes were experienced by no more than 5% of non-cancer controls.
	Urgency	Radiumhemmet, Karolinska University Hospital in Stockholm and Jubileumskliniken, Sahlgrenska University Hospital in Gothenburg<br><br>Treated	Baloch (2021)	Study specific	37%		

Bowel symptom	Indicator	Dataset	First Author (year)	Outcome measure(s)	Prevalence (radiotherapy patients)	Severity (radiotherapy patients)	Comparisons
		1991-2003					
	Bowel urgency	Prostate Cancer Outcomes Study (PCOS) - Surveillance, Epidemiology and End Results (SEER) registries<br><br>Diagnosed 1994-1995	Potosky (2000)	Adapted	Everyday/ some days<br>35.7%		*Comparison of treatments (radiotherapy v other treatments)*<br><br>(odds of having at all)<br>OR (CI) RP v RT 0.4 (0.27, 0.59)<br><br>Bowel urgency was more common among patients treated with radiotherapy than among those treated with surgery.
	Urgent bowel movement	Prostate Cancer Outcomes Study (PCOS) - Surveillance, Epidemiology and End Results (SEER) registries<br><br>Diagnosed 1994-1995	Hamilton (2001)	Study specific	*Almost every day*<br>Before diagnosis 2.2%<br>6 months 7.9%<br>12 months 4.8%<br>24 months 3.5%<br><br>*Some days*<br>Before diagnosis 17.4%<br>6 months 38.8%<br>12 months 31.6%<br>24 months 30.9%<br><br>*Rarely/never*<br>Before diagnosis 79.9%<br>6 months 52.3%<br>12 months 62.8%<br>24 months 64.8%		
	Bowel urgency	Prostate Cancer Outcomes Study (PCOS) - Surveillance, Epidemiology and End Results (SEER) registries<br><br>Diagnosed 1994-1995	Hoffman (2003)	NR[31]	Bowel urgency, RT group:<br>rare/none 68.2%,<br>some days 28.6%,<br>almost every day 3.2%<br><br>Baseline v 24 months $p \leq .0001$		*Comparison of treatments (radiotherapy v other treatments)*<br><br>Bowel urgency, No treatment group:<br>rare/none 83.8%<br>some days 15.9%<br>almost every day 0.2%

Bowel symptom	Indicator	Dataset	First Author (year)	Outcome measure(s)	Prevalence (radiotherapy patients)	Severity (radiotherapy patients)	Comparisons
					Bowel urgency was more common 24 months after pelvic radiotherapy than prior to treatment.		Bowel urgency was more common among patients treated with pelvic radiotherapy than among those who received no treatment.
	Bowel urgency	Prostate Cancer Outcomes Study (PCOS) - Surveillance, Epidemiology and End Results (SEER) registries<br><br>Diagnosed 1994-1995	Potosky (2004)	Adapted	Everyday/ some days<br>28.5%		*Comparison of treatments (radiotherapy v other treatments)*<br><br>OR (CI) RP v EBRT 0.56 (0.36, 0.87)<br><br>Bowel urgency was more common among patients treated with radiotherapy than among those treated with surgery.
	Bowel urgency	Cancer registries of England, Scotland, Wales and Northern Ireland.<br><br>Diagnosed 1995-2015	Downing (2019)	EPIC-26	Moderate or big problem<br>18-42 months post diagnosis<br>EBRT alone<br>11·4% (10·2–12·7)		*Comparison of treatments (radiotherapy v other treatments)*<br><br>Moderate or big problem<br>Surgery alone<br><br>4·4% (3·9–4·9)
	Urgency	Cancer Registry of Norway<br><br>Diagnosed 2004-2015	Fossa (2022)	EPIC-26	% Moderate/big problem<br><br>17% (read from graph)		*Comparison against non-cancer group*<br><br>% Moderate/big problem<br><br>5% (read from graph) No statistical support presented
	Bowel urgency		Resnick (2013)	Adapted	2 years 34%<br>5 years 31.3%<br>15 years 35.8%		*Comparison of treatments (radiotherapy v other treatments)*<br><br>OR (CI) RP v RT<br><br>2 years 0.39 (0.22, 0.68)<br>5 years 0.47 (0.26, 0.84)<br>15 years 0.98 (0.45-2.14)<br><br>Bowel urgency was more common among patients treated with radiotherapy than among those treated with surgery at 2 years and 5

Bowel symptom	Indicator	Dataset	First Author (year)	Outcome measure(s)	Prevalence (radiotherapy patients)	Severity (radiotherapy patients)	Comparisons
							years post-treatment. At 15 years the difference was small and lacked statistical support.
**Bleeding**	Bloody stools	Surveillance, Epidemiology and End Results (SEER) registries<br><br>Diagnosed 2011-2012	Barocas (2017)	EPIC-26			*Comparison of treatments (radiotherapy v other treatments)*<br><br>No difference between EBRT and RP or AS at 6, 12 or 36 months (adjusted)
	Bloody stools	Comparative Effectiveness Analysis of Surgery and Radiation Study (CAESAR) - Surveillance, Epidemiology and End Results (SEER) registries<br><br>Diagnosed 2011-2012	Hoffman (2020)	EPIC-26	% Moderate/big problem<br><br>Baseline 1%<br>6 months 1%<br>1 year 1%<br>3 years 2%<br>5 years 0%		*Comparison of treatments (radiotherapy v other treatments)*<br><br>Comparison EBRT v AS: Odds of moderate or big problem<br>OR (CI) p-value<br><br>6 months 0.8 (0.1, 8.4) p=0.88<br>1 year 0.9 (0.2, 3.5) p=0.9<br>3 years 2.6 (0.3, 21.9) p=0.39<br><br>No clinically meaningful difference between EBRT and AS at any point in time.
	Bloody stools	CEASAR (including small number of CaPSURE) and PCOS), SEER registries<br><br>Diagnosed 2011-2012	Pasalic (2021)	EPIC-26	Moderate or big problem<br><br>EBRT-LDR<br>Baseline 0%<br>6 months 1%<br>1 year 2%<br>3 years 0%<br>5 years 0%<br><br>EBRT<br>Baseline 1%<br>6 months 1%<br>1 year 2%<br>3 years 2%<br>5 years 1%		*Comparison of treatments (radiotherapy)*<br><br>EBRT-LDR v EBRT<br>Effect (adjusted Odds Ratio), (CI), p-value<br><br>6 months 0.5 (0.0,10.3) p=0.64<br>1 year 3.0 (1.2,7.6) p=0.02<br>3 years 0.0 (0.0,0.2) p=0.008<br>5 years 0.0 (0.2,3.3) p=0.003

Bowel symptom	Indicator	Dataset	First Author (year)	Outcome measure(s)	Prevalence (radiotherapy patients)	Severity (radiotherapy patients)	Comparisons
	Bloody stools	Surveillance, Epidemiology and End Results (SEER) registries<br><br>Diagnosed 2011-2012	De (2022)	EPIC-26	Baseline 0%<br>6 months 1%<br>1 year 2%<br>3 years 0%<br>5 years 0%		*Comparison of treatments (radiotherapy v other treatments)*<br><br>EBRT-LDR v RP<br><br>OR (p-value)<br>6 months 1.3 (0.897)<br>1 year 8.4 (<0.001)<br>3 years –<br>5 years -
	Anal leakage of blood while awake	Karolinska University Hospital, Stockholm and Jubileumskliniken, Sahlgren University Hospital, Gothenburg<br><br>Treated<br>1991-2003	Dunberger (2009)	Study specific	7% at least occasionally		*Comparison against non-cancer group*<br><br>RR[32] 2 (1,3.8) comparing with no cancer
							RR 1.6 (1.1-2.2) comparing with no cancer
	Rectal bleeding				17% at least occasionally		Rectal bleeding was more common among patients who had received pelvic radiotherapy than among a non-cancer comparison group.
	Blood discharge	Radiumhemmet, Karolinska University Hospital in Stockholm and Jubileumskliniken, Sahlgrenska University Hospital in Gothenburg<br><br>Treated<br>1991-2003	Steineck (2017)	Study specific	10%		*Comparison against non-cancer group*<br><br>One of 6 syndromes identified by the authors for which the distribution of scores differed between patients and non-cancer comparators, with scores being generally higher among patients. The cut-off for defining the syndrome was such that syndromes were experienced by no more than 5% of non-cancer controls.
	Blood syndrome	Radiumhemmet, Karolinska University Hospital in Stockholm and Jubileumskliniken,	Baloch (2021)	Study specific	13%		

Bowel symptom	Indicator	Dataset	First Author (year)	Outcome measure(s)	Prevalence (radiotherapy patients)	Severity (radiotherapy patients)	Comparisons
		Sahlgrenska University Hospital in Gothenburg<br><br>Treated 1991-2003					
	Blood bother	Cancer Registry of Norway<br><br>Diagnosed 2004-2015	Fossa (2022)	EPIC-26	% Moderate/big problem<br><br>3% (read from graph)		*Comparison against non-cancer group*<br><br>% Moderate/big problem<br><br>1% (read from graph
	Bleeding from anus	Finnish Cancer Registry<br><br>Diagnosed 2004	Lehto (2017)	Study specific	At any time since start of treatment<br>Lasting 3-12 months<br>15%<br>Lasting >12 months<br>59%		
	Bleeding with bowel movement	Prostate Cancer Outcomes Study (PCOS) - Surveillance, Epidemiology and End Results (SEER) registries	Potosky (2004)	Adapted (from earlier PCOS surveys)	EBRT<br>13%		*Comparison of treatments (radiotherapy v other treatments)*<br><br>OR (CI) RP v EBRT 0.58 (0.31, 1.06)<br><br>Patients treated with pelvic radiotherapy were more likely to experience bleeding with bowel movement than those treated with surgery. However, the difference was not statistically significant.
**Diarrhoea**	loose stools distress	All (7) Swedish departments of gynaecological oncology<br><br>Diagnosed 1991-1992	Bergmark (2002)	Study specific	(distress)<br>% much, moderate/much, a little/moderate/much in patients having internal or external RT +/- surgery<br><br>15%, 30%, 42%		*Comparison of treatments (radiotherapy v other treatments)*<br><br>Surgery – much 4%, moderate or much 6%, any 18%<br><br>Distress from loose stools was more prevalent among patients treated with pelvic radiotherapy than those treated with surgery (statistical support not reported).

Bowel symptom	Indicator	Dataset	First Author (year)	Outcome measure(s)	Prevalence (radiotherapy patients)	Severity (radiotherapy patients)	Comparisons
	Diarrhoea	National Cancer Registry Ireland & Northern Ireland Cancer Registry<br><br>Diagnosed 1995-2010	Drummond (2015)	EORTC QLQ-30 and PR-25		Unadjusted mean score EORTC: EBRT+ADT 10.5, EBRT 11.8, BT 6.9	*Comparison of treatments (radiotherapy v other treatments)*<br><br>Multivariate analysis comparing with RP Difference (CI):<br>EBRT+ADT 2.27 (-0.09-4.63)<br>EBRT 3.86 (1.89, 5.82)<br>BT 0.23 (-3.66, 4.11)<br><br>EBRT significantly higher scores than RP - but not clinically significant.
	Diarrhoea	Finnish Cancer Registry<br><br>Diagnosed 2004	Lehto (2017)	Study specific	At any time since start of treatment<br>Lasting 3-12 months 17%<br>Lasting >12 months 32%		
	Diarrhoea	Prostate Cancer Outcomes Study (PCOS) - Surveillance, Epidemiology and End Results (SEER) registries<br><br>Diagnosed 1994-1995	Potosky (2000)	Adapted	Everyday/ some days<br>37.2%		*Comparison of treatments (radiotherapy v other treatments)*<br><br>(odds of having at all)<br>*Comparison of treatments (radiotherapy v other treatments)*<br><br>OR (CI) RP v RT 0.85 (0.46, 1.56)<br><br>Patients treated with pelvic radiotherapy were slightly more likely to experience diarrhoea than those treated with surgery alone. However, the difference was not statistically significant.
	Diarrhoea bother	Prostate Cancer Outcomes Study (PCOS) - Surveillance,	Potosky (2004)	Adapted (from earlier PCOS surveys)	EBRT<br>15.3%		*Comparison of treatments (radiotherapy v other treatments)*

Bowel symptom	Indicator	Dataset	First Author (year)	Outcome measure(s)	Prevalence (radiotherapy patients)	Severity (radiotherapy patients)	Comparisons
	Diarrhoea	Epidemiology and End Results (SEER) registries<br><br>Diagnosed 1994-1995			Everyday/ some days<br>23.3%		OR (CI) RP v EBRT 0.7 (0.41, 1.22)<br><br>Patients treated with pelvic radiotherapy were more likely to experience diarrhoea bother than those treated with surgery. However, the difference was not statistically significant.<br><br>*Comparison of treatments (radiotherapy v other treatments)*<br><br>OR (CI) RP v EBRT 0.85 (0.55, 1.26)<br><br>Patients treated with pelvic radiotherapy were slightly more likely to experience diarrhoea than those treated with surgery alone. However, the difference was not statistically significant.
	Loose stools	Metropolitan Detroit Cancer Surveillance System<br><br>Diagnosed 1996-1998	Schwartz (2002)	Study specific / adapted	T1 6.2%<br>T2 13.8%<br>P=0.041		*Comparison of treatments (radiotherapy v other treatments)*<br><br>Comparison at follow-up of those reporting no bother at baseline<br>RP 14.8%<br>RT 32.1%<br>P=<0.001<br><br>The prevalence of loose stools was greater among patients exposed to pelvic radiotherapy than those treated with surgery (analysis restricted to patients who did not report loose stools at baseline)
	Diarrhoea score	Eindhoven Cancer Registry<br><br>Diagnosed 1999-2007	Van de Poll-Franse (2012)	EORTC QLQ-EN24 plusQLQ-30 item on diarrhoea	16% of EBRT women had "quite a bit" or "very much" diarrhoea	Mean (SD)<br>RT only 17 (28)	*Comparison of treatments (radiotherapy v other treatments)*<br><br>Mean (SD)<br>No LA No RT (N=377) 10 (21) vs LA no RT (N=198) 7 (20) vs RT only (N=153) 17 (28)

Bowel symptom	Indicator	Dataset	First Author (year)	Outcome measure(s)	Prevalence (radiotherapy patients)	Severity (radiotherapy patients)	Comparisons
							vs LA plus RT (N=14) 13 (29) adjusted p-value 0.0002<br><br>Patients treated with pelvic radiotherapy had a higher mean diarrhoea score than those who did not receive pelvic radiotherapy.
**Incontinence**	Faecal leakage distress	All (7) Swedish departments of gynaecological oncology<br><br>Diagnosed 1991-1992	Bergmark (2002)	Study specific	RT – much 10%, moderate or much 13%, any 21%		*Comparison of treatments (radiotherapy v other treatments)*<br><br>Surgery (no RT) (n=93) much 2%, moderate/ much 3%, a little/moderate/much 8%<br><br>Prevalence of faecal leakage distress was greater among patients exposed to pelvic radiotherapy (statistical support for this association was not reported)
	leakage of stools while awake occasionally	Karolinska University Hospital, Stockholm and Jubileumskliniken, Sahlgren University Hospital, Gothenburg<br><br>Treated 1991-2003	Dunberger (2009)	Study specific	33%		*Comparison against non-cancer group*<br><br>RR 6.1 (3.8,9.7) compared with no cancer
	Empty all stools into clothing without warning	Karolinska University Hospital, Stockholm and Jubileumskliniken, Sahlgren University Hospital, Gothenburg<br><br>Treated 1991-2003	Dunberger (2010)	Study specific	12%		*Comparison against non-cancer group*<br><br>Emptying all stools into clothing without forewarning in last 6 months at least occasionally - age adjusted RR 11.9 (3.8,37.8).<br><br>Age adjusted RR increased to:<br>22.1 if exclude survivors and controls with injury to vaginal/perineum,<br>16.1 excluding injury to anal sphincter,<br>16 excluding lactose intolerant.

Bowel symptom	Indicator	Dataset	First Author (year)	Outcome measure(s)	Prevalence (radiotherapy patients)	Severity (radiotherapy patients)	Comparisons
							The experience of faecal incontinence was more common among patients treated with pelvic radiotherapy than among women of similar age in a non-cancer comparison group.
	leakage	Karolinska University Hospital, Stockholm and Jubileumskliniken, Sahlgren University Hospital, Gothenburg<br><br>Treated<br>1991-2003	Steineck (2017)	Study specific	26%		*Comparison against non-cancer group*<br><br>One of 5 syndromes identified by the authors for which the distribution of scores differed between patients and non-cancer comparators, with scores being generally higher among patients. The cut-off for defining the syndrome was such that syndromes were experienced by no more than 5% of non-cancer controls.
	Leakage syndrome	Karolinska University Hospital, Stockholm and Jubileumskliniken, Sahlgren University Hospital, Gothenburg<br><br>Treated<br>1991-2003	Baloch (2021)	Study specific	31%		
	Incontinence	Cancer Registry of Norway<br><br>Diagnosed 2004-2015	Fossa (2022)	EPIC-26	% moderate or big problem<br>7% (read from graph)		*Comparison against non-cancer group*<br><br>% Moderate of big problem<br><br>1% (read from graph) No statistical support presented
	Faecal leakage	Cancer Registry of Norway and Norwegian Prescription Database<br><br>Diagnosed<br>2004	Kyrdalen (2012)	Selected items from EPIC-50	RT 14%<br>RT+HT 14%		*Comparison of treatments (radiotherapy v other treatments)*<br><br>No treatment 3%, RT or RT+HT v No treatment, or RP, p<.01, adjusted for age, risk and comorbidities.<br><br>Prevalence of faecal leakage was statistically higher among patients exposed to pelvic radiation.

Bowel symptom	Indicator	Dataset	First Author (year)	Outcome measure(s)	Prevalence (radiotherapy patients)	Severity (radiotherapy patients)	Comparisons
	(stool) Leakage more than a few drops	Metropolitan Detroit Cancer Surveillance System<br><br>Diagnosed 1996-1998	Schwartz (2002)	Study specific / adapted	T1 1.5%<br>T2 1.5%<br>P=1.00		*Comparison of treatments (radiotherapy v other treatments)*<br><br>Comparison at follow-up of the prevalence of stool leakage among those who reported no bother with stool leakage at baseline<br>RP 0.8%<br>RT 5.5%<br>P=0.038<br><br>The prevalence of stool leakage was greater among patients exposed to pelvic radiotherapy than those treated with surgery (analysis restricted to patients who did not report faecal leakage at baseline)<br><br>Comparison at follow-up of the prevalence of use of pads among those who reported no use of pads at baseline<br>RP 0%<br>RT 4.6%P=0.017<br>The prevalence of pad-use was greater among patients exposed to pelvic radiotherapy than those treated with surgery (analysis restricted to patients who did not report pad-use at baseline)
	Use of pads				T1 0.8%<br>T2 0.8%<br>P=1.00		
**Abdominal pain**	Abdominal pain distress	All (7) Swedish departments of gynaecological oncology<br><br>Diagnosed 1991-1992	Bergmark (2002)	Study specific	(distress)<br>% much, moderate/much, a little/moderate/muc h in patients having internal or external RT +/- surgery		*Comparison of treatments (radiotherapy v other treatments)*<br><br>Surgery alone – much 9%, moderate or much 15%, any 31%<br><br>Patients treated with pelvic radiotherapy were more likely to report distress from abdominal

Bowel symptom	Indicator	Dataset	First Author (year)	Outcome measure(s)	Prevalence (radiotherapy patients)	Severity (radiotherapy patients)	Comparisons
					14%, 27%, 48%		pain than those treated with surgery alone (statistical support not reported).
	Pain	Cancer Registry of Norway<br><br>Diagnosed 2004-2015	Fossa (2022)	EPIC-26	% moderate or big problem<br><br>5% (read from graph)		*Comparison against non-cancer group*<br><br>% moderate or big problem<br><br>3% (read from graph) No statistical support presented
	Pain with bowel movement	Prostate Cancer Outcomes Study (PCOS) - Surveillance, Epidemiology and End Results (SEER) registries<br><br>Diagnosed 1994-1995	Hamilton (2001)	Study specific	*Almost every day*<br>Before diagnosis 2.5%<br>6 months 7%<br>12 months 2.7%<br>24 months 2.1%<br><br>*Some days*<br>Before diagnosis 9.5%<br>6 months 18.6%<br>12 months 10.9%<br>24 months 11.9%<br><br>*Rarely/never*<br>Before diagnosis 87.7%<br>6 months 73%<br>12 months 85.1%<br>24 months 85.1%		
	Painful bowel movement	Prostate Cancer Outcomes Study (PCOS) - Surveillance, Epidemiology and End Results (SEER) registries<br><br>Diagnosed 1994-1995	Potosky (2000)	Adapted	Everyday/ some days<br>13.6%		*Comparison of treatments (radiotherapy v other treatments)*<br><br>(odds of having at all)<br>OR (CI) RP v RT 1 (0.58, 1.8)<br><br>Patients treated with pelvic radiotherapy were slightly more likely to report painful bowel

**Bowel symptom**	**Indicator**	**Dataset**	**First Author (year)**	**Outcome measure(s)**	**Prevalence (radiotherapy patients)**	**Severity (radiotherapy patients)**	**Comparisons**
							movement than those treated with surgery alone. However, the difference was not statistically significant.
	Tenderness during bowel movement	Prostate Cancer Outcomes Study (PCOS) - Surveillance, Epidemiology and End Results (SEER) registries<br><br>Diagnosed 1994-1995	Potosky (2004)	Adapted	Somewhat / a lot<br>EBRT<br>8.4%		*Comparison of treatments (radiotherapy v other treatments)*<br><br>OR (CI) RP v EBRT 0.85 (0.46, 1.56)<br><br>Patients treated with pelvic radiotherapy were slightly more likely to report tenderness during bowel movement than those treated with surgery alone. However, the difference was not statistically significant.<br><br>OR (CI) RP v EBRT 1.31 (0.73, 2.35)<br><br>Patients treated with pelvic radiotherapy were slightly more likely to report painful bowel movement than those treated with surgery alone. However, the difference was not statistically significant
	Painful bowel movement				Everyday/some days<br>EBRT<br>9.4%		
**Painful haemorrhoids**	Painful haemorrhoids	Prostate Cancer Outcomes Study (PCOS) - Surveillance, Epidemiology and End Results (SEER) registries<br><br>Diagnosed 1994-1995	Hamilton (2001)	Study specific	*Almost every day*<br>Before diagnosis 0.8%<br>6 months 2.8%<br>12 months 0.9%<br>24 months 2.9%<br><br>*Some days*<br>Before diagnosis 9.1%<br>6 months 12.3%<br>12 months 10.4%<br>24 months 14.5%		

Bowel symptom	Indicator	Dataset	First Author (year)	Outcome measure(s)	Prevalence (radiotherapy patients)	Severity (radiotherapy patients)	Comparisons
					Rarely/never<br>Before diagnosis 89.8%<br>6 months 84.4%<br>12 months 86.9%<br>24 months 81%<br><br>Baseline v 24 months p≤.005		
	Painful haemorrhoids	Prostate Cancer Outcomes Study (PCOS) - Surveillance, Epidemiology and End Results (SEER) registries<br><br>Diagnosed 1994-1995	Potosky (2000)	Adapted	Everyday/ some days<br>16.3%		*Comparison of treatments (radiotherapy v other treatments)*<br><br>(odds of having at all)<br>OR (CI) RP v RT 0.38 (0.23, 0.64)<br>Patients treated with pelvic radiotherapy were more likely to report painful haemorrhoids than those treated with surgery.
	Painful haemorrhoids	Prostate Cancer Outcomes Study (PCOS) - Surveillance, Epidemiology and End Results (SEER) registries<br><br>Diagnosed 1994-1995	Potosky (2004)	Adapted	Everyday/ some days<br>19.6%		*Comparison of treatments (radiotherapy v other treatments)*<br><br>OR (CI) RP v EBRT 0.43 (0.25, 0.74)<br><br>Patients treated with pelvic radiotherapy were more likely to report painful haemorrhoids than those treated with surgery.
**Rectal wetness**	Rectal wetness	Prostate Cancer Outcomes Study (PCOS) - Surveillance, Epidemiology and End Results (SEER) registries<br><br>Diagnosed 1994-1995	Hamilton (2001)	Study specific	*Almost every day*<br>Before diagnosis 1.8%<br>6 months 4%<br>12 months 1.8%<br>24 months 3.4%<br><br>*Some days*<br>Before diagnosis 10.6%<br>6 months 22.4%<br>12 months 20.5%		

Bowel symptom	Indicator	Dataset	First Author (year)	Outcome measure(s)	Prevalence (radiotherapy patients)	Severity (radiotherapy patients)	Comparisons
					24 months 16.2%<br><br>*Rarely/never*<br>Before diagnosis 87.2%<br>6 months 69.6%<br>12 months 76.2%<br>24 months 79.5%<br><br>Baseline v 24 months p≤.005		
	Rectal wetness	Prostate Cancer Outcomes Study (PCOS) - Surveillance, Epidemiology and End Results (SEER) registries<br><br>Diagnosed 1994-1995	Potosky (2000)	Adapted	Everyday/ some days 2 years post-treatment<br>20.7%		*Comparison of treatments (radiotherapy v other treatments)*<br><br>OR (CI) 0.63 (0.40, 0.99)<br>Patients treated with pelvic radiotherapy were more likely to report rectal wetness than those treated with surgery alone.
	Rectal wetness	Prostate Cancer Outcomes Study (PCOS) - Surveillance, Epidemiology and End Results (SEER) registries<br><br>Diagnosed 1994-1995	Potosky (2004)	Adapted	Everyday/ some days 5 years post-treatment<br>18.3%		*Comparison of treatments (radiotherapy v other treatments)*<br><br>(odds of having at all)<br>OR (CI) RP v RT 0.75 (0.47, 1.20)<br><br>Patients treated with pelvic radiotherapy were more likely to report rectal wetness than those treated with surgery. This difference was not statistically significant.
**Constipation**	Constipation distress	All (7) Swedish departments of gynaecological oncology<br><br>Diagnosed 1991-1992	Bergmark (2002)	Study specific	(distress)<br>% much, moderate/much, a little/moderate/much in patients having internal or external RT +/- surgery<br><br>3%, 8%, 18%		*Comparison of treatments (radiotherapy v other treatments)*<br><br>Surgery alone – much 7%, moderate/much 12%, a little/moderate/much 31%<br><br>Constipation distress was more prevalent among patients treated with surgery than

Bowel symptom	Indicator	Dataset	First Author (year)	Outcome measure(s)	Prevalence (radiotherapy patients)	Severity (radiotherapy patients)	Comparisons
							among those treated with pelvic radiotherapy (statistical support not reported).
	Constipation score	National Cancer Registry Ireland & Northern Ireland Cancer Registry<br><br>Diagnosed	Drummond (2015)	EORTC QLQ-30 and PR-25		Unadjusted mean score EORTC:<br>EBRT+ADT 11.2<br>EBRT 15.6<br>BT 6.0<br>Compared to those who received EBRT with / without concurrent ADT or observation, clinically significantly lower constipation among men treated with BT.	*Comparison of treatments (radiotherapy v other treatments)*<br><br>Multivariate analysis comparing with RP Difference (CI):<br>EBRT+ADT -1.45 (-4.14, 1.25)<br>EBRT 2.57 (0.3, 4.83)<br>BT -2.82 (-7.83-1.74)<br>EBRT significantly higher scores than RP - but not clinically significant.
**Mucous**	mucous from rectum	Prostate Cancer Outcomes Study (PCOS) - Surveillance, Epidemiology and End Results (SEER) registries<br><br>Diagnosed 1994-1995	Potosky (2004)	Adapted	Somewhat/a lot<br><br>*EBRT who reported bother on at least 1 domain*<br><br>13.1%		*Comparison of treatments (radiotherapy v other treatments)*<br><br>OR (CI) RP v EBRT 0.36 (0.20, 0.66)<br><br>Patients treated with pelvic radiotherapy were more likely to report mucous from rectum than those treated with surgery.
	Excessive mucous discharge	Karolinska University Hospital, Stockholm and Jubileumskliniken, Sahlgren University Hospital, Gothenburg<br><br>Treated<br>1991-2003	Steineck (2017)	Study specific	16%		*Comparison against non-cancer group*<br><br>One of 5 syndromes identified by the authors for which the distribution of scores differed between patients and non-cancer comparators, with scores being generally higher among patients. The cut-off for defining the syndrome was such that syndromes were experienced by no more than 5% of non-cancer controls.
**Frequency**	>3 movements per day	Prostate Cancer Outcomes Study (PCOS) - Surveillance,	Hamilton (2001)	Study specific	*Almost every day*<br>Before diagnosis 5.9%<br>6 months 15.3%		

Bowel symptom	Indicator	Dataset	First Author (year)	Outcome measure(s)	Prevalence (radiotherapy patients)	Severity (radiotherapy patients)	Comparisons
		Epidemiology and End Results (SEER) registries<br><br>Diagnosed 1994-1995			12 months 11.9%<br>24 months 7.4%<br>*Some days*<br>Before diagnosis 21%<br>6 months 30.3%<br>12 months 24.3%<br>24 months 26.4%<br>*Rarely/never*<br>Before diagnosis 72.7%<br>6 months 54%<br>12 months 62.4%<br>24 months 65.2%<br><br>Baseline v 24 months post - treatment $p \leq .005$		
**Gas**	Excessive gas discharge	Karolinska University Hospital, Stockholm and Jubileumskliniken, Sahlgren University Hospital, Gothenburg<br><br>Treated	Steineck (2017)	Study specific	15%		*Comparison against non-cancer group*<br><br>One of 5 syndromes identified by the authors for which the distribution of scores differed between patients and non-cancer comparators, with scores being generally higher among patients. The cut-off for defining the syndrome was such that syndromes were experienced by no more than 5% of non-cancer controls.

Table is arranged in descending order of frequency of papers reporting each bowel symptom, beginning with the symptom reported in the most papers. Heavy horizontal lines indicate divisions between different bowel symptoms. Bands of shading /no shading group papers of the same dataset reporting on that symptom. Within groups, papers are arranged in chronological order by publication date and then alphabetical order by first author.

[1]Comparative Effectiveness of Surgery and Radiotherapy, [2]Cancer of the Prostate Strategic Urologic Research Endeavor, [3]Surveillance Epidemiology and End Results, [4]Expanded Prostate Index Composite, [5]Confidence Interval, [6]External Beam Radiotherapy, [7]Active Surveillance, [8]Radical Prostatectomy, [9]Difference in Difference, [10]University of California Los Angeles Prostate Cancer Index, [11]salvage Radiotherapy, [12]Inter Quartile Range, [13]Low Dose Rate, [14]Radiotherapy [15]Standard Deviation, [16]Conformal Radiotherapy, [17]Hyperfractionated Radiotherapy, [18]Pelvis Only-Intensity Modulated Radiotherapy, [19]Pelvis and Lymph Node - Intensity Modulated Radiotherapy, [20]Brachytherapy Boost, [21]High Dose Radiotherapy, [22]Androgen Deprivation Therapy, [23]European Organisation for Research and Treatment of Cancer, [24]Quality of Life Questionnaire, [25]Prostate Cancer, [26]Brachytherapy, [27]Hormonal Therapy [28]Watchful Waiting [29]Endometrial Cancer [30]Lymphoadectomy, [31]Odds Ratio, [32]Not Reported, [33]Relative Risk
